# Machine Learning‐Assisted Evaluation of Circulating DNA Quantitative Analysis for Cancer Screening

**DOI:** 10.1002/advs.202000486

**Published:** 2020-07-29

**Authors:** Rita Tanos, Guillaume Tosato, Amaelle Otandault, Zahra Al Amir Dache, Laurence Pique Lasorsa, Geoffroy Tousch, Safia El Messaoudi, Romain Meddeb, Mona Diab Assaf, Marc Ychou, Stanislas Du Manoir, Denis Pezet, Johan Gagnière, Pierre‐Emmanuel Colombo, William Jacot, Eric Assénat, Marie Dupuy, Antoine Adenis, Thibault Mazard, Caroline Mollevi, José María Sayagués, Jacques Colinge, Alain R. Thierry

**Affiliations:** ^1^ IRCM Institut de Recherche en Cancérologie de Montpellier INSERM U1194 Montpellier F‐34090 France; ^2^ Institut régional du Cancer de Montpellier Montpellier F‐34298 France; ^3^ Université de Montpellier Montpellier F‐34090 France; ^4^ CHU Lapeyronnie Montpellier F‐34090 France; ^5^ Faculty of Science II Department of Biochemistry Lebanese University Fanar Beirut 2611 Lebanon; ^6^ CHU Clermont‐Fd Clermont‐Ferrand 63100 France; ^7^ CHU Saint‐Eloi Montpellier F‐34090 France; ^8^ Centro de Investigación del Cáncer Cytometry General Service and Department of Medicine University of Salamanca Salamanca 37007 Spain

**Keywords:** cancer, circulating DNA, early diagnosis, machine learning, screening

## Abstract

While the utility of circulating cell‐free DNA (cfDNA) in cancer screening and early detection have recently been investigated by testing genetic and epigenetic alterations, here, an original approach by examining cfDNA quantitative and structural features is developed. First, the potential of cfDNA quantitative and structural parameters is independently demonstrated in cell culture, murine, and human plasma models. Subsequently, these variables are evaluated in a large retrospective cohort of 289 healthy individuals and 983 patients with various cancer types; after age resampling, this evaluation is done independently and the variables are combined using a machine learning approach. Implementation of a decision tree prediction model for the detection and classification of healthy and cancer patients shows unprecedented performance for 0, I, and II colorectal cancer stages (specificity, 0.89 and sensitivity, 0.72). Consequently, the methodological proof of concept of using both quantitative and structural biomarkers, and classification with a machine learning method are highlighted, as an efficient strategy for cancer screening. It is foreseen that the classification rate may even be improved by the addition of such biomarkers to fragmentomics, methylation, or the detection of genetic alterations. The optimization of such a multianalyte strategy with this machine learning method is therefore warranted.

## Graphical abstract

Certain circulating DNA structural characteristics enable the distinction between plasma from healthy and cancer individuals. Using this observation, a machine learning decision tree prediction model is implemented for noninvasive early detection. The high sensitivity and specificity of the test on more than 1000 individuals supports the belief in the need for machine learning assistance when combining quantitative and/or multiple biomarkers.

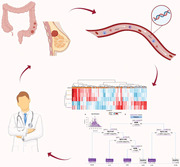

## 1 Introduction

Cancer biomarkers exist to monitor the response to treatment for a specific cancer or group of cancers. They are limited, however, with regard to sensitivity and specificity in early detection or mass screening.^[^ ^1^ ^]^

Machine learning methods were applied for the development of predictive models,^[^ ^2^ ^]^ such as artificial neural networks (ANNs), Bayesian networks, support vector machines (SVMs), and decision trees (DTs). Different strategies have been described for the detection of different cancer types,^[^ ^3^ ^]^ e.g., by identifying a subset of three single nucleotide polymorphisms as key discriminators between control and breast cancer patients.^[^ ^4^ ^]^

The use of cfDNA^[^ ^5^ ^]^ was recently proposed by our team, among others,^[^ ^6^, ^7^ ^]^ for cancer screening and early detection. Existing approaches include the detection of the viral DNA of the Epstein‐Barr virus in asymptomatic nasopharyngeal cancer cases,^[^ ^8^ ^]^ to the analysis of the methylome and cfDNA methylation patterns in different cancer types.^[^ ^9^, ^10^ ^]^ cfDNA levels^[^ ^11^ ^]^ and the detection of genetic alterations^[^ ^12^, ^13^ ^]^ have also been considered for their diagnostic potential. Other studies have associated mutation detection and protein markers.^[^ ^6^ ^]^ Despite intensive research, however, the reliability of cfDNA‐based tests have been impacted by issues regarding their sensitivity and specificity, especially for the early stages of cancer.

Our team (among others) has worked for more than a decade on the quantitative and structural characteristics of cfDNA,^[^ ^14^, ^15^, ^16^, ^17^, ^18^ ^]^ as a means of discriminating cancer and healthy subjects. We have identified various quantitative and structural biomarker candidates, and have independently evaluated their performance in three experimental models. We subsequently proposed a machine learning method based on a predictive DT model in a large cohort of healthy and cancer individuals. We believe that cfDNA‐based attempts at developing a pan‐cancer test should be multianalyte, using a combination of qualitative (i.e., presence or absence of genetic or epigenetic alterations) and quantitative parameters. In order to rigorously evaluate the predictive potential of different combinations of quantitative parameters, therefore, we are convinced that building our machine learning method's proof of concept is warranted.

## 2 Results

### 2.1 Determination of cfDNA Quantitative Parameters in a Xenografted Mouse Model

**Figure 1:**
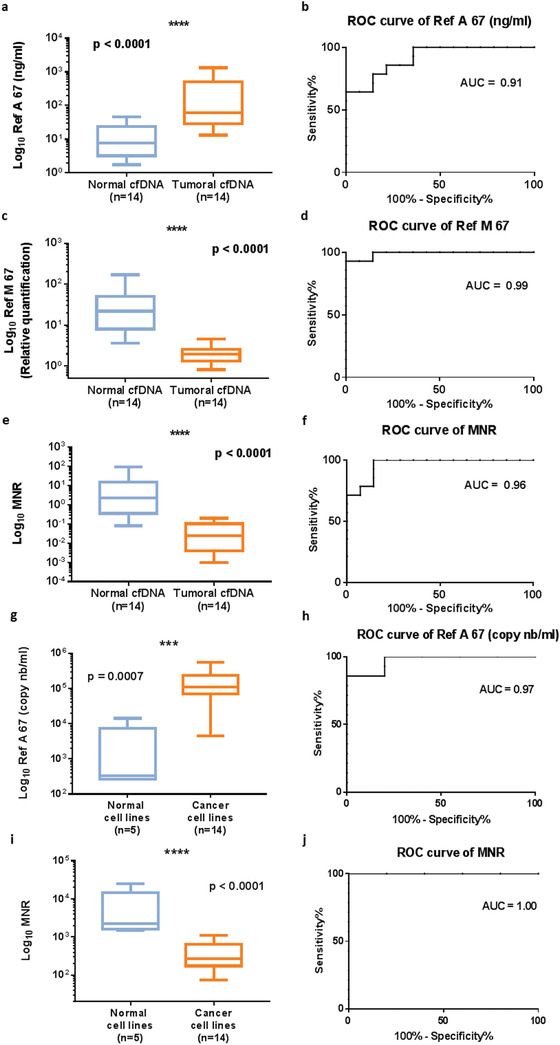
Efficiency of total nuclear and mitochondrial cfDNA amount in discriminating tumor and normal DNA in a xenografted mouse model, and tumor and normal cells in culture. a) Total nuclear cfDNA amount (Ref A 67; ng mL^−1^ of plasma) of normal (murine) or tumor (human) origin in 14 SW620 xenografted nude mice. b) ROC curve of tumor versus nontumor Ref A 67 (ng mL^−1^) in mouse model. c) Relative quantification of mitochondrial cfDNA (Ref M 67) of normal or tumor origin in mouse model. d) ROC curve of tumor versus nontumor Ref M 67 in mouse model. e) Mitochondrial to nuclear cfDNA ratio (MNR) as determined in normal and tumor cfDNA in mouse model. f) ROC curve from normal and tumor MNR in mouse model. g) Boxplot of the total nuclear cfDNA concentration (Ref A 67 copy number mL^−1^ of supernatant) for normal and cancer cell lines. h) ROC curve for Ref A 67 between normal and cancer cell lines. i) Boxplot of the MNR for normal and cancer cell lines. j) ROC curve for MNR between normal and cancer cells. The nonparametric Wilcoxon–Mann–Whitney test was used to compare the different parameters. The boxplot whiskers represent the minimal and maximal value; *p*: *p*‐value, probability value; ROC: receiver operating characteristics; AUC: area under curve; MNR: mitochondrial to nuclear ratio.

To determine the potential of different quantitative cfDNA parameters, we used a murine model of nude mice xenografted with SW620 colorectal cancer human cells which we had established previously.^[^ ^15^ ^]^ This model allowed us to differentiate human tumor cfDNA from normal murine cfDNA in the same mouse. Using Q‐PCR (quantitative polymerase chain reaction), we quantified nuclear and mitochondrial cfDNA of human and murine origin in 14 xenografted mice. We found a significant increase of total nuclear DNA concentrations (ng mL^−1^) (which we called Ref A 67), in tumor over normal cfDNA. The receiver operating characteristics (ROC) curve analysis showed an AUC (area under curve) of 0.91 (0.8125–1.014, 95% CI; confidence interval) (**Figure** **1**a,b) (Sensitivity Se = 0.79; Specificity Sp = 0.86). The results were also significant when analyzing mitochondrial DNA concentrations (Ref M 67) (Figure 1c,d) and the mitochondrial to nuclear ratio (MNR) (Figure 1e,f). These two parameters decreased significantly and featured higher AUCs of 0.99 (0.963–1.016, 95% CI) and 0.96 (0.9047–1.024, 95% CI), with respective sensitivities of 0.93 and 1, and specificities of 1 and 0.86. This suggests the high potential that mitochondrial DNA concentration and the MNR have for distinguishing between cfDNA of normal and of tumor origin.

### 2.2 Evaluation of the Ref A 67 and the MNR in Cell Culture Supernatants

The Ref A 67 and the MNR were then tested in the supernatant of cells in culture, to assess their ability to discriminate between normal and cancer cells. The supernatant of 14 cancer and 5 normal cell lines were tested (Table S1, Supporting Information). The Ref A 67 showed a significant increase, with a high AUC of 0.97 (0.901–1.042, 95% CI) (Se = 0.86, Sp = 1) (Figure 1g,h), while the MNR showed a potential of 100% in discriminating between normal and cancer cells in this series of 19 cell lines (Figure 1i,j).

### 2.3 Evaluation in an Exploratory Cohort of Healthy and Cancer Subjects

Next, the quantitative parameters were validated in the plasma of a small independent exploratory cohort of 76 healthy individuals and 50 stage IV colorectal cancer (CRC) patients (**Table** **1**). The Ref A 67 showed a significant increase in CRC patients with an AUC of 0.81 (0.73–0.89, 95% CI) (Figure S1a,b, Supporting Information). As for the Ref M 67, a significant decrease was observed in cancer patients with an AUC of 0.89 (0.83–0.95, 95% CI) (Figure S1c,d, Supporting Information). The MNR then showed a decrease, with the highest AUC (AUC = 0.98; 0.96–0.99, 95% CI) between healthy subjects and stage IV CRC patients (Figure S1e,f, Supporting Information).

**Table 1:** Patients’ characteristics

				Gender			Age	
	Diagnosis group		Nb	Male	Female	NA	Mean	Median
Exploratory cohort	Colorectal cancer	Stage IV	50	25 (50%)	25 (50%)	–	65	65
	Healthy		76	48 (63%)	28 (37%)	–	51	53
Confirmation cohort	Colorectal cancer	All stages	791	452 (57%)	331 (42%)	8 (1%)	70	70
		Stages 0/I/II	425	252 (59.3%)	172 (40.5%)	1 (0.2%)	72	72
		Stage III	180	108 (60%)	72 (40%)	–	70	71
		Stage IV	186	92 (49%)	87 (47%)	7 (4%)	65	66
	Breast cancer	Stages II or III	169	–	169 (100%)	–	49	48
	Other cancers	HCC	18	18 (100%)	–	–	62	64
		Pancreatic	3	2 (67%)	1 (33%)	–	78	78
		Ovarian	1	–	1 (100%)	–	44	44
		Lymphoma	1	1 (100%)	–	–	69	69
		Total	23	21 (91%)	2 (9%)	–	63	66
	All cancers		983	473 (48.1%)	502 (51.1%)	8 (0.8%)	66	67
	Healthy		289	179 (62%)	110 (38%)	–	43	46

a, Nb: number; b, NA: not available; c, HCC: Hepatocellular carcinoma.

### 2.4 Validation of the cfDNA Parameters in a Large Retrospective Cohort

The discriminative value of these cfDNA quantitative parameters was then evaluated in a large retrospective cohort of 289 healthy individuals and 983 patients with CRC (*N =* 791), breast (*N =* 169), or other types of cancers (hepatocellular, pancreatic, ovarian, and lymphoma) (*N =* 23) (Table 1). The total nuclear cfDNA concentration showed a significant increase, with a *p*‐value of < 0.0001 between healthy individuals and the different cancer types (CRC, breast and others) (Figure S2a, Supporting Information). This was the case between the healthy group and CRC patients, regardless of their stage (stage 0/I/II, stage III, stage IV) (Figure S2b, Supporting Information). An AUC of 0.83 (0.81–0.86, 95% CI) was observed for the CRC group compared to healthy subjects (Figure S3a, Supporting Information), with a high value of 0.79 (0.76–0.83, 95% CI) for early stages 0, I, and II (Figure S3b, Supporting Information). The Ref A 67 showed an AUC of 0.7 (0.74–0.82, 95% CI) for the discrimination of the breast cancer group from the healthy group (Figure S3c, Supporting Information).

We then assessed the potential of the MNR. This parameter decreased significantly between healthy patients and all cancer types (Figure S2c, Supporting Information). It showed a good discriminative potential for CRC patients (AUC = 0.78; 0.75–0.82, 95% CI) and breast cancer patients (AUC = 0.82; 0.78–0.86, 95% CI) (Figure S4a,c, Supporting Information). A significant decrease and a high discriminative potential were also observed between the healthy group and early stage CRC patients (AUC = 0.79; 0.76–0.82, 95% CI) (Figures S2d and S4b, Supporting Information).

### 2.5 Cohort Age Adjustment and cfDNA Parameters’ Individual Performance for CRC Detection

**Figure 2:**
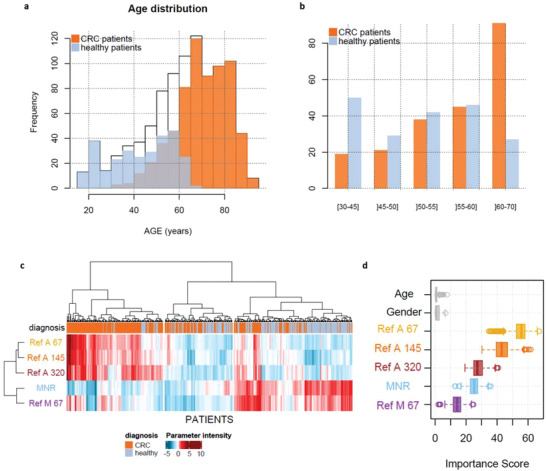
Representation of patients used for bootstrapping and age correction. a) Age distribution among all patients (white) and the two sub‐populations: patients with colorectal cancer (orange) and healthy patients (blue). b) Categories based on patients’ age, in order to select one patient with colorectal cancer with one healthy patient. c) Ascending hierarchical clustering, performed on all patients used for resampling and all parameters available. d) Boxplot of each parameter importance score obtained after 500 recursive partitioning regression trees and resampling.

With the aim of maximizing discriminative power, we wanted to assess the potential of a machine learning approach, by evaluating the combination of different cfDNA variables. We first focused on the CRC group compared to healthy subjects. A bias was observed in the age distribution of the studied cohort (**Figure** **2**a); the fraction of patients < 30 years old was constituted of healthy individuals only, and the fraction of patients >70 years old of CRC patients only. No gender bias was observed (Figure S5, Supporting Information). For this reason, subjects between 30 and 70 years old were regrouped in five categories: ≤ 45 years old, 45–50, 51–55, 56–60, and > 60 years old (Figure 2b). The tendency of all parameters was then appreciated on a heatmap (Figure 2c) clustering patients (by columns) and parameters (by rows). The importance score of each parameter was then extracted, after the application of recursive partitioning on 500 different resampling (Figure 2d).

**Figure 3:**
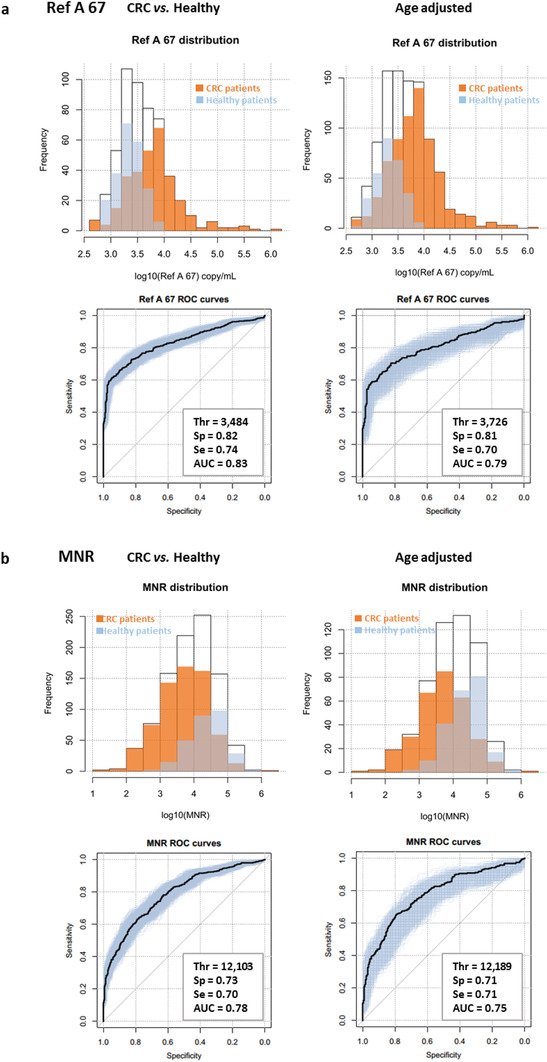
Distribution and ROC curves of a) the Ref A 67 (copy nb mL^−1^) and b) MNR parameters among all patients (white), CRC patients (orange), and healthy individuals (blue), before and after age adjustment.

The descriptive statistics of the CRC cohort are presented in Table S2 in the Supporting Information. The different quantitative cfDNA parameters were assessed: Ref A 67 (copy nb mL^−1^) or total nuclear cfDNA concentration, Ref M 67 (copy nb mL^−1^) or total mitochondrial cfDNA concentration, and the MNR. The age‐adjusted estimation of the AUC for the Ref A 67 (copy nb mL^−1^) parameter decreased slightly between healthy and CRC patients compared to the nonadjusted value: 0.79 (0.73–0.84, 95% CI) for the age‐adjusted AUC with 0.81 Sp (0.72–0.89, 95% CI) and 0.70 Se (0.62–0.78, 95% CI) versus 0.83 AUC (0.81–0.86, 95% CI), 0.82 Sp (0.77–0.87, 95% CI), and 0.74 Se (0.69–0.78, 95% CI) for the nonadjusted cohort (**Figure** **3**a and **Table** **2**). The MNR showed an AUC of 0.75 (0.69–0.81, 95% CI) after age resampling with 0.71 Sp (0.61–0.781, 95% CI) and 0.7 Se (0.62–0.78, 95% CI) (Figure 3b and Table 2). The total mitochondrial cfDNA concentration (Ref M 67 ng mL^−1^ plasma) did not have a very high discriminative potential, with a relatively low AUC value after resampling of 0.61 (0.53–0.67, 95% CI); Sp = 0.60 (0.45–0.69, 95% CI), Se = 0.62 (0.52–0.78, 95% CI) (Figure S6, Supporting Information and Table 2).

**Table 2:** Parameters estimation for each variable in the CRC cohort

	Threshold		Specificity		Sensitivity		AUC	
Parameter	Estimation	IC95%	Estimation	IC95%	Estimation	IC95%	Estimation	IC95%
CRC versus healthy								
Ref A 67	3484	[3076–4857]	0.82	[0.77–0.87]	0.74	[0.69–0.78]	0.83	[0.81–0.86]
Ref A 145	1491	[1121–1639]	0.79	[0.70–0.86]	0.74	[0.69–0.82]	0.83	[0.80–0.86]
Ref A 320	425	[286–509]	0.77	[0.69–0.83]	0.67	[0.62–0.74]	0.76	[0.73–0.79]
MNR	12 103	[6945–15 360]	0.73	[0.65–0.82]	0.70	[0.61–0.76]	0.78	[0.75–0.82]
Ref M 67	4.41E+07	[2.78E+07–6.04E+07]	0.60	[0.46–0.69]	0.57	[0.49–0.69]	0.59	[0.56–0.63]
Patients paired by age category CRC versus healthy								
Ref A 67	3726	[2921–4511]	0.806	[0.72–0.89]	0.7	[0.62–0.78]	0.786	[0.73–0.84]
Ref A 145	16 113	[1267–1874]	0.793	[0.67–0.91]	0.715	[0.62–0.84]	0.794	[0.74–0.87]
Ref A 320	617	[100–899]	0.763	[0.67–0.92]	0.631	[0.53–0.73]	0.631	[0.53–0.73]
MNR	12 189	[4295–16 225]	0.713	[0.61–0.81]	0.707	[0.59–0.82]	0.748	[0.69–0.81]
Ref M 67	4.78E+07	[3.54E+07–6.87E+07]	0.602	[0.45–0.69]	0.618	[0.52–0.78]	0.606	[0.53–0.67]
Patients paired by age category early stages (0‐I‐II) versus healthy								
Ref A 67	3415	[1800–4321]	0.747	[0.57–0.91]	0.64	[0.49–0.80]	0.694	[0.58–0.82]
Ref A 145	1542	[1012–1874]	0.753	[0.57–0.92]	0.672	[0.53–0.84]	0.725	[0.62–0.85]
Ref A 320	502	[72–706]	0.719	[0.54–0.90]	0.651	[0.49–0.82]	0.689	[0.58–0.81]
MNR	15 022	[6960–21 942]	0.676	[0.51–0.84]	0.664	[0.51–0.84]	0.668	[0.55–0.78]
Ref M 67	4.71E+07	[1.16E+07–7.13E+07]	0.639	[0.43–0.81]	0.576	[0.40–0.74]	0.571	[0.45–0.68]

a, Parameters estimation for each variable based on minimum distance between corresponding ROC curve and the point (Se = 1, 1‐Sp = 0) with the use of an empirical bootstrap to build their respective confidence interval. These estimations were obtained before and after age adjustment with a focus on early stages (0‐I‐II) colorectal cancer patients. b, Se = sensitivity; c, Sp = specificity; d, AUC = area under curve.

Two other cfDNA parameters were studied in the plasma of the majority of the tested cohort, which were to be added to the quantitative variables. These parameters correspond to the nuclear cfDNA concentration of the fragments with a size ≥ 145 base pairs (bp) (Ref A 145 (copy nb mL^−1^ plasma)) or ≥ 320 bp (Ref A 320 (copy nb mL^−1^ plasma)). They provide information regarding the size distribution of cfDNA in the healthy and the cancer group. The Ref A 145 was significantly higher in CRC patients than in healthy individuals, with a high discriminative value (age‐adjusted AUC = 0.79 (0.74–0.87, 95% CI); Sp = 0.79 (0.67–0.91, 95% CI), Se = 0.72 (0.62–0.84, 95% CI) (Figure S7, Supporting Information and Table 2). The AUC of the Ref A 320 was shown to be lower than that of the Ref A 145: AUC = 0.63 (0.53–0.73, 95% CI) with 0.76 Sp (0.67–0.92, 95% CI) and 0.63 Se (0.53–0.73, 95% CI) (Figure S8, Supporting Information and Table 2).

Regarding early stage (0‐I‐II) CRC patients versus healthy individuals, when taken individually these parameters showed lower sensitivity, specificity, and AUC, with much larger confidence intervals than those obtained when analyzing CRC patients of all stages (Table 2).

### 2.6 DT Construction and Prediction Value for CRC Detection

**Figure 4:**
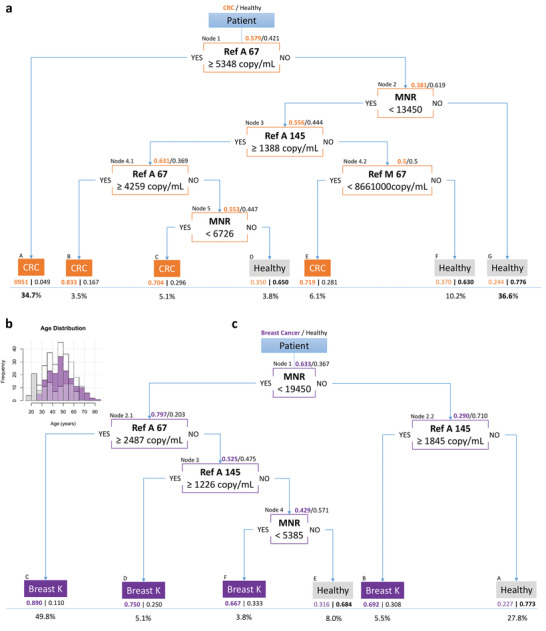
Global DTs obtained from the colorectal cancer and breast cancer cohorts. a) Global DT obtained from the colorectal cancer cohort by iterating a recursive partitioning regression tree after resampling patients at each step. Proportions of patients with colorectal cancer (orange) and healthy patients (gray) are represented at each node. b) Distribution histogram of the ages of patients (white), in breast cancer patients (purple) and in healthy patients (gray). c) Global DT obtained from the breast cancer cohort by iterating a recursive partitioning regression tree after resampling patients at each step. Proportions of patients with breast cancer (purple) and healthy patients (gray) are represented at each node. Each final leaf in each tree contains the name of the most representative population inside, with their relative proportions. The proportion of patients that ended in each leaf is presented below the corresponding leaf. One node features the parameter of interest, the condition on the parameter (below), and the direction of the answer (YES/NO).

We implemented a DT prediction model by including our five different cfDNA quantitative and structural parameters, obtained from Q‐PCR analysis after age resampling, to examine whether their association would further increase the discriminative power (**Figure** **4**a and Table S3, Supporting Information). 527 CRC and healthy patients were used for the construction of the tree, and showed 0.87 specificity (0.79–0.92, 95% CI) and 0.76 sensitivity (0.67–0.85, 95% CI) (**Table** **3**). Performance was assessed by the cross‐validation of 2000 rounds of resampling of a total of 424 patients not used for the tree construction. This validation estimated a specificity of 0.89 (0.84–0.94, 95% CI) and a sensitivity of 0.77 (0.73–0.80, 95% CI) for CRC and healthy patients’ classification (Table 3). Our DT prediction model provides improved specificity and sensitivity, compared to discrimination made using only individual parameters (Table 2). When applied to early stages CRC, a relatively high Sp and Se of 0.87 (0.83–0.91, 95% CI) and 0.72 (0.67–0.76, 95% CI), respectively, were observed (Table 3), improving the quality and robustness of early stages CRC detection.

**Table 3:** Estimation of tree performances over several groups of patients, obtained from the colorectal and breast cancer cohorts

Specificity		Sensitivity	
Estimation	95% CI	Estimation	95% CI
CRC decision tree			
Training set—Patients used for tree building *N* = 527 (CRC + healthy)			
0.87	[0.79–0.92]	0.76	[0.67–0.85]
Testing set—Patients not used for tree building *N* = 424 (CRC + healthy)			
0.89	[0.84–0.94]	0.77	[0.73–0.80]
CRC stages 0/I/II versus healthy			
0.87	[0.83–0.91]	0.72	[0.67–0.76]
Breast cancer patients *N* = 278 versus healthy			
0.90	[0.84–0.95]	0.58	[0.50–0.65]
Breast cancer decision tree			
Training set—Patients used for tree building *N* = 236 (breast cancer + healthy)			
0.72	[0.65–0.84]	0.86	[0.80–0.91]
Testing set—Patients not used for tree building *N* = 46 (breast cancer + healthy)			
0.80	[0.64–0.95]	0.95	[0.85–1]
CRC patients *N* = 951 versus healthy			
0.66	[0.62–0.72]	0.85	[0.82–0.87]

a, The confidence intervals were obtained using the empirical bootstrap method.

### 2.7 Breast Cancer and Other Cancer Types

The distribution and discriminatory power of each of our five cfDNA parameters were tested in breast cancer patients (*N* = 169) of stages II and III versus healthy individuals. A two‐sample nonparametric one‐sided Mann–Whitney test with normal approximation (*n* > 50) was adopted for continuous variables comparison of the populations studied (Figure S9 and Table S4, Supporting Information). The MNR and the Ref A 67 showed the highest discriminatory power, with an AUC of 0.81 (0.76–0.87, 95% CI) and 0.77 (0.71–0.83, 95% CI), a sensitivity of 0.76 (0.66–0.85, 95% CI) and 0.75 (0.66–0.86, 95% CI) and a specificity of 0.75 (0.66–0.85, 95% CI) and 0.77 (0.61–0.81, 95% CI), respectively (Figure S10 and Table S5, Supporting Information). The other three parameters were less effective in discriminating breast cancer patients from healthy individuals (Ref M 67: AUC = 0.72 (0.66–0.88, 95% CI), Se = 0.67 (0.50–0.77, 95% CI), Sp = 0.71 (0.59–0.85, 95% CI); Ref A 145: AUC = 0.69 (0.62–0.76, 95% CI), Se = 0.61 (0.44–0.76, 95% CI), Sp = 0.71 (0.53–0.89, 95% CI); Ref A 320: AUC = 0.60 (0.53–0.70, 95% CI), Se = 0.55 (0.34–0.65, 95% CI), Sp = 0.66 (0.52–0.86, 95% CI)) (Figure S10 and Table S5, Supporting Information). In the DT construction, we adopted the same strategy for the breast cancer cohort (Figure 4b,c and Table S3, Supporting Information) as we had for the CRC cohort. The MNR parameter appeared at the first node, followed by the Ref A 67 and the Ref A 145. We used 236 patients in total for the construction of the tree, with a specificity of 0.72 (0.65–0.84, 95% CI) and a sensitivity of 0.86 (0.80–0.91, 95% CI) (Table 3). Cross‐validation was done using the 46 breast cancer and healthy patients not used for the tree construction, and showed a high specificity of 0.80 (0.64–0.95, 95% CI) and a high sensitivity of 0.95 (0.85–1, 95% CI) (Table 3). The predictive tree established on one cohort was then used to predict the results of the other. By applying the breast cancer cohort on the CRC DT, we observed a high specificity of 0.90 (0.84–0.95, 95% CI) but a poor sensitivity of 0.58 (0.50–0.65, 95% CI). The breast cancer DT showed a low specificity of 0.66 (0.62–0.72, 95% CI) but a high sensitivity of 0.85 (0.82–0.87, 95% CI) for the prediction of CRC patients (Table 3). We hypothesized that the algorithm may be cancer‐type specific. The ability of these two DTs for the classification of 23 patients with different types of cancers (pancreatic, hepatocellular, lymphoma, and ovarian) was assessed. 21 and 22 individuals out of the 23 were classified as cancer patients by the CRC and the breast prediction tree, respectively (Table S6, Supporting Information).

## 3 Conclusion

Despite significant statistical differences in cfDNA concentration, the large overlap in cancer and healthy subjects^[^ ^17^, ^19^ ^]^ severely undermines its potential as a tool for cancer screening. Alternatively, another strategy which has been considered is the detection of genetic alterations from cfDNA.^[^ ^6^, ^12^, ^20^, ^21^ ^]^ Indeed, several attempts at this have recently been implemented. However, the false positive rates observed preclude its application, mainly due to i) the detection of white blood cell‐derived mutations among cfDNA variants (notably with age), and ii) the very low circulating tumor DNA (ctDNA) rate in a significant number of cancer patient plasma samples. We hypothesized that quantitative and structural indicators from total cfDNA not completely and directly associated with malignancy may compensate the eventual deficiencies of qualitative indicators, such as genetic or epigenetic alterations, especially with respect to sensitivity. We believe that the combination of both types of measures in a multianalyte approach might meet the stringent levels of reliability required for the evaluation of a screening test or an early detection test. This study aims to provide the proof of concept of using cfDNA quantitative biomarkers for such tests, and proposes a machine learning method developed specifically to that end.

Drawing on our extensive work on the origins and structures of cfDNA, in particular from cancer patients, we have identified two quantitative cfDNA candidate parameters which can be used to discriminate healthy individuals and cancer patients: the Ref A 67 and the MNR, determined by specific Q‐PCR, targeting both nuclear and mitochondrial sequences. In this study, we first validated these two parameters independently, in three experimental models: cell culture media, tumor xenografted mice, and plasma from an exploratory cohort of healthy and cancer subjects.

Both our own team's work^[^ ^16^, ^17^ ^]^ and that of others^[^ ^22^, ^23^, ^24^ ^]^ have shown that cfDNA fragmentation^[^ ^25^ ^]^ and size profiles differ in healthy and cancer patients, especially in the fractions below 60 bp, between 60 and 145 bp, between 145 and 300 bp, and higher than 300 bp.^[^ ^16^, ^26^, ^27^ ^]^ For this reason, we added two parameters to our study: the Ref A 145 (nuclear cfDNA concentration of the fragments with a size ≥ 145 base pairs) and the Ref A 320 (≥ 320 bp), which provide information regarding cfDNA size distribution. The identification of these parameters originated from previous reports showing that the examination of size profile and fragmentomics^[^ ^25^ ^]^ may help in discriminating healthy and cancer individuals.^[^ ^16^, ^26^, ^27^ ^]^ Subsequently, these variables were all evaluated in a large retrospective cohort of 289 healthy individuals and 983 patients with CRC (*N* = 791), breast (*N* = 169), and other types of cancers (hepatocellular, pancreatic, ovarian, and lymphoma) (*N* = 23). This evaluation was done using a machine learning approach, with the variables considered both independently and in combination, after age resampling. Total nuclear cfDNA concentration increases in cancer patients compared to healthy individuals, but mitochondrial cfDNA levels are reduced. Although the cellular level of mitochondria in cancer patients is now under intense scrutiny, there remain significant discrepancies in the literature. Furthermore, little is known about the production or release of mitochondrial cfDNA into the blood stream. This makes it difficult currently to provide a definitive explanation for this observation. The Ref A 67 and the MNR showed the two highest AUCs, and therefore the highest potential for discriminating cancer patients and healthy individuals.

The implementation of a DT prediction model for the detection and classification of healthy and cancer patients showed very encouraging results even for early CRC stages (specificity of 0.89 (0.84–0.94; 95% CI) and sensitivity of 0.72 (0.67–0.76; 95% CI)). It also showed an increase in discrimination potential. These results emphasize the importance of integrating both the nuclear and mitochondrial origin of cfDNA. Each of the tested parameters was selected as a discriminating variable at a node of the DT model, except for the Ref A 320. Although a difference can be discerned in the Ref A 320 levels of colorectal cancer and healthy controls, e.g., in Figure S8 in the Supporting Information, Ref A 320 has shown itself to be correlated with Ref A 67 and Ref A 145. As a result, the tree construction algorithm never selected Ref A 320 as the most discriminant variable for any node.

Among the many techniques available for automatic classification, we opted for a robust, high‐performance method which is capable of revealing nonlinear relationships between variables, and whose output would be exploited in a clinical setting in the future. For these reasons, we decided to use recursive regression trees,^[^ ^28^ ^]^ which compared favorably to other machine learning techniques, such as ANNs or SVM for mortality prediction in gliomas.^[^ ^29^ ^]^ However, the conventional deployment of regression trees with cross‐validation (2/3 as learning set, 1/3 as test set), such as in the discrimination between head and neck squamous cell carcinoma patients and healthy adults,^[^ ^30^ ^]^ produced unstable results from our data. We obtained excessively large confidence intervals for sensitivity and specificity (Table S7 and Figure S11, Supporting Information), and the resulting DTs were highly dependent on the training set. The main reason for this was the age bias in our data, which contained an overrepresentation of cancer and healthy cases among older and younger patients, respectively. In addition, we noted that age was related to other parameters in a nonlinear way (Table S8, Supporting Information). We therefore divided patients into age classes, in such a way that an equivalent number of cancer and healthy cases could be drawn from each class before being pooled for training and testing. To obtain a robust DT, we applied an iterative procedure where trees were first generated by multiple resampling of the data (with correction for age bias), and the variable to be used as the most recurrent top node was fixed. A second round of resampling yielded a new series of trees, with the latter variable at the top; that variable was then used to select the most recurrent next level nodes, etc. At every step, i.e., for every node/variable, the threshold was estimated with a confidence interval which was built using a bootstrap method. This type of iteration has been applied in other domains, e.g., to infer suicide attempts^[^ ^31^ ^]^ or to detect drug usage.^[^ ^32^ ^]^ The final tree was tested on the patients not used for training. The iterative construction procedure resulted in more stable trees with narrower confidence intervals than direct regression (Table S7 and Figure S11, Supporting Information).

The selected machine learning method appears well suited to classifying and testing combinations of quantitative biomarkers. This would also encourage its use for combinations of both quantitative and qualitative biomarkers. The selected parameters evaluated in this study showed a significant screening power, but by themselves were not sufficiently efficient and robust to distinguish early stage patients. Their combination in a DT for the CRC or the breast cancer cohort revealed better results, and showed an improvement over the CancerSeek test^[^ ^6^ ^]^ and over the analysis of the cfDNA methylation profile.^[^ ^9^ ^]^

Breast cancer patients were poorly predicted by the DT based on the CRC cohort (Se = 0.58), despite the high specificity found (0.90). This high specificity could be explained by the fact that the same healthy patients were included for the construction of both algorithms. Alternatively, the use of the breast cancer tree to classify colorectal cancer patients was quite efficient (Se = 0.85), although the drop in specificity (0.66) was notable. This could be due to the fact that the breast cancer cohort only concerns women, and that a difference probably exists between healthy males and females regarding the cfDNA measured parameters, especially for mitochondrial cfDNA.^[^ ^33^ ^]^ This may also explain the importance of the MNR, which appears in the first node of the breast cancer DT, for the classification of breast cancer patients. The algorithm was then applied on a small cohort of patients with other cancer types (*N* = 23), and showed a good level of prediction given the sensitivity. This seems to indicate that these trees are not cancer‐type specific.

The reliability of biomarkers based on the total cfDNA quantification is also known to vary in conditions and pathologies other than cancer, such as autoimmune diseases,^[^ ^34^ ^]^ sepsis,^[^ ^35^ ^]^ myocardial infraction,^[^ ^36^ ^]^ exercise,^[^ ^37^ ^]^ and others. It is therefore crucial to investigate the impact on this approach of confounding conditions (such as inflammation). Although the quantitative multianalyte test performance presented in this work is noteworthy as a blood test, our study presents critical limitations in the context of a universal cancer screening. The studied cohort is retrospective and composed of healthy controls devoid of asymptomatic patients. While it does appear to confirm diagnosis at the pre‐screening stage, for it to be properly called “screening” the test ought to have been performed on an asymptomatic population ostensibly in good health, with positive results being confirmed by other testing techniques.

Protein markers have approximately a > 10% sensitivity and a > 99% specificity, and have the capacity to indicate one or more cancer types.^[^ ^7^ ^]^ However, their diagnostic use does not currently meet the requirements for multicancer tests used at the population scale. The primary requirements for this are test performance (high sensitivity without sacrifice of specificity; > 90% and > 99%, respectively), reproducibility, and robustness. In addition, cancer screening test validation should ultimately be subject to large‐scale population studies of people with no known diagnosis, with the inclusion of potentially confounding conditions in order to ensure specificity, and using multiple study sites to ensure demographic diversity. We believe that a blood test based on cfDNA analysis might have the capacity to achieve this goal, especially given its minimal invasiveness, high compliance, affordability, and scalability.

Other requirements for such a test would be generalizability to population, and the capacity to identify anatomic location, in order to direct appropriate diagnostic follow up. The biomarkers tested in this study cannot determine the tumor tissue of origin, which is one of the reported potentials of CancerSeek and methylation analysis.^[^ ^6^, ^9^ ^]^ For this reason, the addition of other parameters might improve the predictive capacity of the model. The detection of genetic alterations such as mutations, aneuploidy, or translocations/re‐arrangements would a priori seem to be the best screening parameters. However, these qualitative indicators showed some limitations as screening biomarkers. This is because current methods implying cfDNA analysis, such as conventional next generation sequencing (NGS), show some restrictions in obtaining useful data, especially in the plasma of patients with a small tumor.^[^ ^6^ ^]^ In addition, white blood cell‐derived mutations among cfDNA variants (especially with age) might lead to false positive results.^[^ ^38^ ^]^ For these reasons, their use was precluded as a single strategy.^[^ ^39^ ^]^

A cfDNA‐based cancer screening test would be multianalyte. Although the performance of the combination of quantitative and structural parameters as reported here is not of a sufficient standard, such a combination may reveal synergic power, especially with respect to early cancer stage detection, when associated with cfDNA parameters from different origins, such as genetic alterations. An examination of genome‐wide methylation profiling was found to be specific to cancer and very informative as to cancer type, while also showing good sensitivity and specificity.^[^ ^9^ ^]^ This could therefore be a candidate partner of choice. Analysis of the cfDNA fragment size profile might be another powerful candidate, as we have previously demonstrated,^[^ ^26^, ^27^ ^]^ and as has been recently confirmed.^[^ ^40^, ^41^ ^]^ The synergistic value of using our approach in combination with another cancer detection method should therefore be assessed, in order to confirm its additive advantage.

This study validated the concept of using a DT based on cfDNA quantitative and structural parameters to distinguish cancer patients from healthy individuals. The ultimate goal would be to use machine learning to combine these parameters with others, such as methylation profile and fragmentomics, in order to achieve a better selectivity between different cancer types in the context of pan‐cancer testing. Evaluation of the combination of the quantitative biomarkers tested here with methylation and fragmentomics analysis is ongoing, using the method we have proposed.

## 4 Experimental Section

##### Study Design

This study presented an observational analysis of different quantitative and structural cfDNA parameters, calculated using an ultrasensitive Q‐PCR method to quantify various wild type nuclear and mitochondrial cfDNA sequences in the plasma of cancer patients and healthy individuals. To establish its proof of concept, this study also proposed a machine learning method based on a predictive DT model combining these different parameters for early cancer detection.

The plasma of a small exploratory cohort of 76 healthy subjects and 50 CRC patients (stage IV) was first analyzed. Then 1272 plasma samples (Table 1) of 289 healthy individuals and 983 other patients with CRC (*N =* 791), breast (*N =* 169), or other cancer types (hepatocellular, pancreatic, ovarian, and lymphoma) (*N =* 23) were analyzed over a range of stages. Early stages CRC patients (stages 0, I, and II) represented more than 50% of the total CRC cohort tested.

The capacity of each of the tested cfDNA parameters was evaluated to discriminate between healthy individuals and cancer patients, especially for early stages cancers. Then a DT was developed, which combined five different parameters, and established its sensitivity and specificity, in order to assess its potential for early cancer detection.

##### Patient and Sample Characteristics

Blood samples from healthy individuals were obtained from healthy donors, from the Etablissement Français du Sang (E.F.S), which is Montpellier's blood transfusion center (Convention EFS‐PM N° 21PLER2015‐0013). These samples were analyzed (virology, serology, immunology, blood numeration) and ruled out whenever any abnormality was detected.

Plasma samples from patients with colorectal cancer were provided by the hospital centers of Clermont‐Ferrand and Limoges in France, and of Salamanca in Spain, as well as from the “BCB colon” cohort from Montpellier in France (ICM Biobank (Biobank number BB‐0033‐00059)) and from the KPLEX I and KPLEX II (ClinicalTrials.gov Identifier: NCT02784639) studies previously conducted by the team. Plasma samples from individuals with breast cancer were obtained from the “IDEA Sein” study (ClinicalTrials.gov Identifier: NCT03255486) provided by the ICM Biobank (Biobank number BB‐0033‐00059) of Montpellier. Hepatocellular carcinoma plasma samples were provided by Eric Assenat and Marie Dupuy from Montpellier, and three pancreatic adenocarcinomas, one lymphoma, and one ovarian cancer samples were obtained from the hospital center of Salamanca in Spain. All plasma samples from cancer patients were obtained at the time of diagnosis, or at least 45 days after treatment interruption to eliminate any bias due to the liberation of cfDNA by the treatment. Written informed consent was obtained from all participants prior to the onset of the study. The characteristics of all patients included are listed in Table 1.

##### Cell Lines

The supernatant of 14 tumor cell lines in culture was analyzed (Table S1, Supporting Information): three colorectal cancer (SW620, SW480, and CaCo2), five prostate cancer (VCAP, 22rV1, DU145, LNCAP, and PC3), three breast cancer (SUM159, MDA 468, and R2sh P53), two lymphoma (RAMOS and BJAB), and one lung cancer cell line (H1975). Five normal cell lines of various origins were also analyzed: human foreskin fibroblasts (HFF), skin fibroblasts (CDC45K), mammary fibroblasts (R2), lung fibroblasts (IMR‐90 A), and hepatocytes (LWFD). Genomic DNA of the DIFI human colorectal cancer cell line grown in RPMI 1640 and supplemented with 10% fetal calf serum and ATB (antibiotics) was used as a standard for human nuclear DNA quantification. Most cell lines were obtained from the American type culture collection (Manassas, VA, USA). The R2sh P53 cell line was obtained from the Charles Theillet/Claude Sardet team. Cell culture medium supernatant was collected when cells were at 70% confluence, and underwent two centrifugations: the first at 1200 *g*, and the second at 16 000 *g*, at 4 °C for 10 min each. DNA was then extracted using the QIAamp DNA Blood Mini Kit (Qiagen, Courtaboeuf, France) from 200 µL of the supernatant and eluted in a final volume of 80 µL.

##### Mice Models

An experimental xenografted mouse model developed previously was used,^[^ ^15^ ^]^ to allow to discriminate within the same mouse the nontumor‐derived cfDNA (of murine origin) and tumor‐derived cfDNA (of human origin). Mice were purchased from Harlan (Gannat, France) and maintained in a specific pathogen‐free facility in an accredited establishment (N° B‐34‐172‐27; Institut de Recherche en Cancérologie de Montpellier‐CRLC Val d'Aurelle‐Paul Lamarque, Montpellier, France). Peripheral blood was drawn into ethylenediaminetetraacetic acid (EDTA) pre‐coated tubes of 14 female athymic nude mice (6–8 weeks old) xenografted with 1 × 10^6^ SW620 human cancer cells 30 days post‐injection, and was used for plasma preparation within 1 h. Tumors were also collected and weighed (200–600 g). All experiments complied with the current national and institutional regulations and ethical guidelines, and were performed by an accredited person (Dr. B. ROBERT, N°34‐156). Research projects involving the use of animals were authorized by the French Ministry of Research.

##### Plasma Isolation and cfDNA Extraction

Human and murine blood samples were handled according to an established preanalytical guideline.^[^ ^33^, ^42^ ^]^ Blood was drawn in EDTA tubes and centrifuged at 1200 *g* at 4 °C in a Heraeus Multifuge LR centrifuge for 10 min. The supernatants were isolated and centrifuged at 16 000 *g* at 4 °C for 10 min. Subsequently, the plasma was either immediately handled for DNA extraction or stored at −80 °C. cfDNA was extracted from 200 µL of plasma and eluted in a final volume of 80 µL using the QIAamp DNA Blood Mini Kit (Qiagen, Courtaboeuf, France) according to the published protocol.^[^ ^43^ ^]^ DNA samples were kept at −20 °C until use. Freeze–thaw cycles were avoided in order to reduce the phenomenon of cfDNA fragmentation. Hemolyzed blood samples were discarded and not included in the study. Hemolysis did not affect cfDNA, but was an indicator of mishandling or improper storage of sample tubes which had led to blood cell deterioration.

##### DNA Quantification by Q‐PCR and Copy Number Calculation

Q‐PCR amplifications were carried out at least in triplicate, in a 25 µL reaction volume on a CFX96 touch Real‐Time PCR detection system (Bio‐Rad) instrument, using the CFX manager software. Each PCR reaction mixture was composed of 12.5 µL of SsoAdvanced Universal SYBR Green Supermix (Bio‐Rad, Marnes‐la‐Coquette, France), 2.5 µL of each amplification primer (3 pmol µL^−1^), 2.5 µL of Nuclease free water (Qiagen), and 5 µL of DNA extract. Thermal cycling was consisted of three repeated steps: a 3 min Hot‐start polymerase activation–denaturation step at 95 °C, followed by 40 repeated cycles at 90 °C for 10 s, then at 60 °C for 30 s. Melting curves were obtained by increasing the temperature from 55 to 105 °C, with a plate reading every 0.2 °C. The concentration was calculated from the Cq detected by Q‐PCR. A triplicate of nontemplate negative control was included in each run for each pair of primers.

##### Human DNA Quantification

For human nuclear DNA, serial dilutions of genomic DNA from the DIFI human colorectal cancer cells were used as a standard for nuclear DNA quantification. The initial concentration and purity were assessed by optic density at *λ* = 260, 230, and 280 nm, with an Eppendorf BioPhotometer D30.

A control standard curve using a 3382 bp human ORF (open reading frame) vector with a 786 bp *MT‐CO3* insert obtained from ABM good (accession no.YP_003024032) of known concentration was used for human mitochondrial DNA quantification in cell lines and human samples. Initial vector solution concentration and purity were determined by measuring the optic density at *λ* = 260, 230, and 280 nm, with an Eppendorf BioPhotometer D30.

For each human plasma sample and cell line supernatant extract, a nuclear wild type *KRAS* amplicon and a *MT‐CO3* mitochondrial amplicon of 67 bp each were separately quantified. A wild type *BRAF* nuclear amplicon of 105 bp was also targeted for several human plasma samples in order to validate the total nuclear DNA quantification, which was calculated by targeting the *KRAS* 67 bp amplicon. *KRAS*, *BRAF*, and *MT‐CO3* were monogenic, and the respective targeted sequences were known to be wild type, and not affected by any mutation or genetic alteration. *KRAS* sequence‐based detection was always controlled by *BRAF* sequence‐based detection, and both quantifications had previously shown a very high correlation.^[^ ^17^, ^33^ ^]^ Targeting these specific genes confirmed whether the cfDNA fraction quantified was derived from the nucleus or the mitochondria. It was previously demonstrated that quantification of both mitochondrial and nuclear Q‐PCR systems was highly specific, down to 1 copy/reaction (Ref A 67 and Ref M 67).^[^ ^33^ ^]^ Amplicons of 145 and 320 bp of the KRAS gene were also targeted for cfDNA size profile analysis. Based on the team's work,^[^ ^16^, ^17^ ^]^ as well the work of others,^[^ ^22^, ^23^, ^24^ ^]^ it was shown that cfDNA was present in the blood mainly in the form of mononucleosomes, highlighting a chromatin organization and that fragmentation^[^ ^25^ ^]^ and size profiles were differed in healthy and cancer patients, especially in the fractions between 60 and 145 bp, between 145 and 300 bp, and higher than 300 bp.^[^ ^16^, ^26^, ^27^ ^]^ As mononucleosomes had a size of 145–168 bp, the following fractions based on the Q‐PCR systems were quantified: the total amount of cfDNA or Ref A 67 (amplicon of 67 bp); the amount of fragments over the mononucleosome size or Ref A 145 (amplicon of 145 bp); and the amount of cfDNA over the mononucleosome size or Ref A 320 (320 bp). The Q‐PCR primers system used to amplify DNA sequences of various sizes was previously validated by the team, and demonstrated high specificity.^[^ ^17^, ^38^ ^]^ It should also be noted that this system was recently compared to whole‐genome sequencing of single‐stranded DNA library preparation, and that both analytical approaches showed similar results regarding the size distribution of cfDNA in cancer patients. That similarity could be taken as a confirmation of the system's accuracy.^[^ ^18^ ^]^

*Nuclear cfDNA copy number calculation*: Nuclear cfDNA copy number per milliliter of plasma/supernatant was determined with the following calculation

(1) $$Q_{\mathrm{nuclear}}=\left(\frac{c}{3.3}\right)*\left(\frac{V_{\mathrm{elution}}}{V_{\mathrm{plasma}/\mathrm{supernatant}}}\right)$$

*Q*_nuclear_ is the NcfDNA copy number per milliliter; *c* is the NcfDNA concentration (pg µL^−1^), determined by Q‐PCR targeting the nuclear *KRAS* gene sequence; 3.3 pg is the human haploid genome mass; *V*_elution_ is the volume of cfDNA extract (µL); and *V*_plasma/supernatant_ is the volume of plasma or supernatant used for the extraction (mL).

*Mitochondrial cfDNA copy number calculation*: Mitochondrial cfDNA copy number per milliliter of plasma/supernatant was determined with the following calculation

(2) $$Q_{\mathrm{mito}}=\left(\frac{c*N_{a}}{2*\mathrm{MW}*\;L_{\mathrm{vector}}}\right)*\left(\frac{V_{\mathrm{elution}}}{V_{\mathrm{plasma}/\mathrm{supernatant}}}\right)$$

*Q*_mito_ is the McfDNA copy number per milliliter of plasma/supernatant; *c* is the McfDNA mass concentration (g µL^−1^) determined by Q‐PCR targeting the mitochondrial *MT‐CO3* gene; *N*_a_ is Avogadro's number (6.02 × 10^23^ molecules per mole); *L*_vector_ is the plasmid length (nucleotides); MW is the molecular weight of one nucleotide (g mol^−1^); *V*_elution_ is the elution volume of cfDNA extract (µL); and *V*_plasma_ is the volume of plasma or supernatant used for the extraction (mL).

##### Murine DNA Quantification

For murine samples, murine nuclear DNA was quantified using as reference a serial dilution of genomic murine DNA (Promega). Human nuclear DNA of tumor origin was quantified using a standard curve of genomic DNA of the human Difi cell line. The relative amount of mitochondrial DNA to nuclear DNA was determined using the equation 2^−*dCq*^, where *dCq* = (*Cq* mito – *Cq* nuc). Human cfDNA (of tumor origin) and murine cfDNA (of nontumor origin) were quantified in the same murine plasma samples using adequate primer sets for each amplification, and expressed in nanograms per milliliter of plasma (ng mL^−1^). Human *KRAS* nuclear amplicon and *MT‐CO3* mitochondrial amplicon of 67 bp each were quantified to assess the human cfDNA of tumor origin. On the other hand, murine cfDNA of nontumor origin was analyzed by targeting a *KRAS* murine nuclear amplicon of 63 bp and a *MT‐CO1* murine mitochondrial amplicon of 114 bp.

##### Oligonucleotides

Oligonucleotides were synthesized and purified by high performance liquid chromatography by Integrated DNA Technologies (Coralville, Iowa). Quality control of the oligonucleotides was performed by matrix‐assisted laser desorption/ionization‐time of flight. They were tested for specificity and sensitivity before use. The sequences and characteristics of the selected primers are presented in Table S9 in the Supporting Information. The primer selection and validation were done in accordance with stringent internal guidelines, requiring the use of four distinct software programs developed by the team.

##### *Ref A, Ref M, and MNR Calculation—*Human Samples and Cell Culture Supernatant Extracts

The mean nuclear DNA copy nb mL^−1^ value when targeting the *KRAS* wild type 67 bp amplicon was named Ref A 67 (copy nb mL^−1^ of plasma or supernatant), and corresponded to the total concentration of nuclear cfDNA. Plasma samples with a Ref A 67 below 450 copies mL^−1^ were not included in the analyzed cohorts. The Ref A 145 (copy nb mL^−1^) and the Ref A 320 (copy nb mL^−1^) were corresponded to the concentrations of cfDNA fragments > 145 bp and > 320 bp, respectively, and were calculated by targeting the KRAS 145 bp or 320 bp amplicon. An additional level of quality control was also used. This consisted of calculating the cfDNA integrity index for each sample DII (DII = Ref A 320/Ref A 67). When the DII was high (> 0.4), the sample was excluded from the analysis, as this suggested that the fraction of long cfDNA fragments was high, and that the sample could be contaminated by genomic DNA derived from white blood cells.

The mean mitochondrial DNA copy nb mL^−1^ value when targeting the *MT‐CO3* wild type 67 bp amplicon was named Ref M 67 (copy nb mL^−1^ of plasma or supernatant), and corresponded to the total concentration of mitochondrial cfDNA.

The MNR was expressed as the ratio of the mean of mitochondrial DNA copy nb mL^−1^ of plasma (or supernatant) value to the Ref A 67 (copy nb mL^−1^ of plasma (or supernatant)) value of the experiments (MNR = Ref M 67/Ref A 67).

##### *Ref A, Ref M, and MNR Calculation—*Murine Samples

For murine samples, human tumor MNR was expressed as the ratio of human mitochondrial cfDNA relative concentration to human nuclear cfDNA concentration (ng mL^−1^). Murine nontumor MNR was expressed as the ratio of murine mitochondrial cfDNA relative concentration to murine nuclear cfDNA concentration (ng mL^−1^).

##### Statistical Analysis

*Murine, cell culture supernatant, and exploratory cohort samples*: Data were expressed as mean ± SD. The nonparametric Wilcoxon–Mann–Whitney test was used for the comparison of medians for murine, cell culture supernatant, and exploratory cohort data. ROC curve was presented and the AUC was calculated. A probability of less than 0.05 was considered to be statistically significant; *p ≤ 0.05, **p ≤ 0.01, ***p ≤ 0.001, ****p ≤ 0.0001.

*Human plasma samples*: The analysis was conducted using R 3.6.0 and the *stats* package.

*Colorectal cancer cohort*: Patients with missing values on parameters used for tree regression were removed. This concerned 131 patients (129 CRC patients and 2 healthy patients). A two‐sample nonparametric one‐sided Mann–Whitney test with normal approximation (*n* > 50) was adopted for continuous variables comparisons among the populations studied, rather than a two‐sample proportion test with continuity correction for binary variables comparison (Table S2, Supporting Information).

*Breast cancer cohort*: Patients with missing values on parameters used for tree regression were removed. This concerned two patients diagnosed with breast cancer. A two‐sample nonparametric one‐sided Mann–Whitney test with normal approximation (*n* > 50) was adopted for continuous variables comparison among the populations studied (Table S4, Supporting Information).

*Age adjustment*: Due to different age distribution among healthy and colorectal cancer patients (Figure 2a), age was split into five different categories in order to pair patients: less than 45 years old, 45 to 50 years old, 51 to 55 years old, 56 to 60 years old, and more than 60 years old (Figure 2b). This division into categories reduced the number of patients included to 527. Moreover, interaction effect between age and some measured parameters was observed using logistic modeling of the colorectal cancer status probability (Table S8, Supporting Information), which confirmed the benefit of removing this bias. This age distribution between breast cancer patients and healthy individuals was equally accentuated, and resulted in the overrepresentation of young healthy patients. Accordingly, a window of patients between 25 and 65 years was created.

*Univariate preliminary analysis*: For each measured parameter, ROC curves were produced using the pROC package. An optimal threshold corresponding to the best prediction was estimated by minimizing the distance between the ROC curve and the ideal point (Se = 1, 1‐Sp = 0). Sensitivity, specificity, AUC, and the optimal threshold were estimated with their respective confidence intervals using an empirical bootstrap method, whose use for quantitative diagnostic tests had already been demonstrated.^[^ ^44^ ^]^ First, 2000 resampling were applied with replacement and the punctual values were estimated for each step. The difference between each step estimation and the targeted value were then calculated, with the 2.5th and 97.5th distance percentiles being obtained and added to the targeted punctual value. Second, the same method of 2000 resampling was applied to 155 CRC and 155 healthy patients among the previously established age categories. Third, the same method was applied to patients from the breast cancer cohort, with 2000 resampling of 236 patients.

*Machine learning*: The cancer prediction algorithm was conducted by applying recursive partitioning for regression trees^[^ ^45^ ^]^ using the *rpart* package. First, a global view of all the measured parameters was generated, in order to appreciate the tendency of each one, by ascending hierarchical clustering. The resulting heatmap was built using the ComplexHeatmap package (Figure 2c), clustering patients (by columns), and parameters (by rows), based on the Euclidian distance and using the ward.D2 agglomeration method. The values were reduced among the samples following log_10_‐transformation, in order to appreciate the variability in a convenient color scale. Second, recursive partitioning (using the *rpart* package) was applied to 500 different resampling, in order to extract the importance score of each variable introduced in the procedure (Figure 2d). This allowed to exclude some variables created by certain parameter combinations (data not shown). It also allowed to check the absence of bias regarding the *Age* and *Gender* variables.

To build the global tree, an iterative procedure was performed on each node to retain the most frequently selected variable; this was done using the resampling method described in the *Univariate Preliminary Analysis* section above. The results were confirmed by applying a homogeneity *Χ* ^2^‐test to the frequency table. Also, a proportion test with continuity correction was applied to the two most frequent variables, in order to discriminate them. Once the selection was confirmed, the parameter of interest at the node was fixed, and its conditional threshold was estimated using the median calculated on 2000 resamplings with replacement. The confidence interval was obtained using the distribution of the difference from the mean. The median was kept as the condition applied at the node according to empirical bootstrap methods (Table S3, Supporting Information). Branch expansion was stopped as soon as i) no specific parameter could split the patients further, ii) less than 10% of patients in the learning set would remain upon further splitting, and iii) no parameter was selected according to an *X*² test. This procedure was iterated with a display in order to obtain fully visual trees for both the colorectal (Figure 4a) and breast cancer cohorts (Figure 4b,c). Each node contained the variable with its estimated threshold and the proportions of cancer/healthy patients present at this step. Each leaf contained the inferred status (cancer or healthy), the proportion of cancer/healthy patients, and the global proportion of patients ending at this leaf. For each DT obtained, its performance was assessed by comparing the predictive values given by the tree and the real diagnosis. Sensitivity and specificity were punctually estimated, then the 95% confidence interval was subsequently obtained by empirical bootstrap, as described in the *Univariate Preliminary Analysis* section above. Different groups of patients were used. First, the parameters were calculated with the patients used for tree building (*N* = 527 for CRC, *N* = 236 for breast cancer). Second, the tree prediction was applied to the remaining patients not included in the tree building. These predictions were also applied to early (0‐I‐II) CRC patients (Table 3).

*Other cancer types*: These parameters were measured using 23 patients with different cancer types. The results were predicted using both the breast cancer cohort tree and the CRC cohort tree (Table S6, Supporting Information).

## Conflict of Interest

The authors declare no conflict of interest.

## Supporting information

Supporting Information

## References

[1] L. D. Maxim , R. Niebo , M. J. Utell , Inhalation Toxicol. 2014, 26, 811. 10.3109/08958378.2014.955932 PMC4389712 25264934

[2] K. Kourou , T. P. Exarchos , K. P. Exarchos , M. V. Karamouzis , D. I. Fotiadis , Comput. Struct. Biotechnol. J. 2015, 13, 8. 25750696 10.1016/j.csbj.2014.11.005 PMC4348437

[3] L. Hussain , A. Ahmed , S. Saeed , S. Rathore , I. A. Awan , S. A. Shah , A. Majid , A. Idris , A. A. Awan , Cancer Biomarkers 2018, 21, 393. 29226857 10.3233/CBM-170643 PMC13078284

[4] J. Listgarten , S. Damaraju , B. Poulin , L. Cook , J. Dufour , A. Driga , J. Mackey , D. Wishart , R. Greiner , B. Zanke , Clin. Cancer Res. 2004, 10, 2725. 15102677 10.1158/1078-0432.ccr-1115-03

[5] A. R. Thierry , S. El Messaoudi , P. B. Gahan , P. Anker , M. Stroun , Cancer Metastasis Rev. 2016, 35, 347. 27392603 10.1007/s10555-016-9629-x PMC5035665

[6] J. D. Cohen , L. Li , Y. Wang , C. Thoburn , B. Afsari , L. Danilova , C. Douville , A. A. Javed , F. Wong , A. Mattox , R. H. Hruban , C. L. Wolfgang , M. G. Goggins , M. Dal Molin , T.‐L. Wang , R. Roden , A. P. Klein , J. Ptak , L. Dobbyn , J. Schaefer , N. Silliman , M. Popoli , J. T. Vogelstein , J. D. Browne , R. E. Schoen , R. E. Brand , J. Tie , P. Gibbs , H.‐L. Wong , A. S. Mansfield , J. Jen , S. M. Hanash , M. Falconi , P. J. Allen , S. Zhou , C. Bettegowda , L. A. Diaz , C. Tomasetti , K. W. Kinzler , B. Vogelstein , A. M. Lennon , N. Papadopoulos , Science 2018, 359, 926. 29348365 10.1126/science.aar3247 PMC6080308

[7] R. Tanos , A. R. Thierry , Transl. Cancer Res. 2018, 7, S105.

[8] K. C. A. Chan , J. K. S. Woo , A. King , B. C. Y. Zee , W. K. J. Lam , S. L. Chan , S. W. I. Chu , C. Mak , I. O. L. Tse , S. Y. M. Leung , G. Chan , E. P. Hui , B. B. Y. Ma , R. W. K. Chiu , S.‐F. Leung , A. C. van Hasselt , A. T. C. Chan , Y. M. D. Lo , N. Engl. J. Med. 2017, 377, 513. 28792880 10.1056/NEJMoa1701717

[9] S. Y. Shen , R. Singhania , G. Fehringer , A. Chakravarthy , M. H. A. Roehrl , D. Chadwick , P. C. Zuzarte , A. Borgida , T. T. Wang , T. Li , O. Kis , Z. Zhao , A. Spreafico , T. da S. Medina , Y. Wang , D. Roulois , I. Ettayebi , Z. Chen , S. Chow , T. Murphy , A. Arruda , G. M. O'Kane , J. Liu , M. Mansour , J. D. McPherson , C. O'Brien , N. Leighl , P. L. Bedard , N. Fleshner , G. Liu , M. D. Minden , S. Gallinger , A. Goldenberg , T. J. Pugh , M. M. Hoffman , S. V. Bratman , R. J. Hung , D. D. De Carvalho , Nature 2018, 563, 579. 30429608 10.1038/s41586-018-0703-0

[10] A. A. I. Sina , L. G. Carrascosa , Z. Liang , Y. S. Grewal , A. Wardiana , M. J. A. Shiddiky , R. A. Gardiner , H. Samaratunga , M. K. Gandhi , R. J. Scott , D. Korbie , M. Trau , Nat. Commun. 2018, 9, 4915. 30514834 10.1038/s41467-018-07214-w PMC6279781

[11] N. Krishnamurthy , E. Spencer , A. Torkamani , L. Nicholson , J. Clin. Med. 2017, 6, 3. 10.3390/jcm6010003 PMC5294956 28054963

[12] J. Phallen , M. Sausen , V. Adleff , A. Leal , C. Hruban , J. White , V. Anagnostou , J. Fiksel , S. Cristiano , E. Papp , S. Speir , T. Reinert , M.‐B. W. Orntoft , B. D. Woodward , D. Murphy , S. Parpart‐Li , D. Riley , M. Nesselbush , N. Sengamalay , A. Georgiadis , Q. K. Li , M. R. Madsen , F. V. Mortensen , J. Huiskens , C. Punt , N. van Grieken , R. Fijneman , G. Meijer , H. Husain , R. B. Scharpf , L. A. Diaz , S. Jones , S. Angiuoli , T. Ørntoft , H. J. Nielsen , C. L. Andersen , V. E. Velculescu , Sci. Transl. Med. 2017, 9, eaan2415. 28814544 10.1126/scitranslmed.aan2415 PMC6714979

[13] L. Fernandez‐Cuesta , S. Perdomo , P. H. Avogbe , N. Leblay , T. M. Delhomme , V. Gaborieau , B. Abedi‐Ardekani , E. Chanudet , M. Olivier , D. Zaridze , A. Mukeria , M. Vilensky , I. Holcatova , J. Polesel , L. Simonato , C. Canova , P. Lagiou , C. Brambilla , E. Brambilla , G. Byrnes , G. Scelo , F. Le Calvez‐Kelm , M. Foll , J. D. McKay , P. Brennan , EBioMedicine 2016, 10, 117. 27377626 10.1016/j.ebiom.2016.06.032 PMC5036515

[14] A. Thierry , S. El Messaoudi (INSERM), EU 15816218.0 – 1111 / 3209797, 2016.

[15] A. R. Thierry , F. Mouliere , C. Gongora , J. Ollier , B. Robert , M. Ychou , M. Del Rio , F. Molina , Nucleic Acids Res. 2010, 38, 6159. 20494973 10.1093/nar/gkq421 PMC2952865

[16] F. Mouliere , B. Robert , E. A. Peyrotte , M. Del Rio , M. Ychou , F. Molina , C. Gongora , A. R. Thierry , PLoS One 2011, 6, e23418. 21909401 10.1371/journal.pone.0023418 PMC3167805

[17] F. Mouliere , S. El Messaoudi , D. Pang , A. Dritschilo , A. R. Thierry , Mol. Oncol. 2014, 8, 927. 24698732 10.1016/j.molonc.2014.02.005 PMC5528519

[18] C. Sanchez , M. W. Snyder , R. Tanos , J. Shendure , A. R. Thierry , npj Genomic Med. 2018, 3, 31. 10.1038/s41525-018-0069-0 PMC6251887 30479833

[19] H. Schwarzenbach , D. S. B. Hoon , K. Pantel , Nat. Rev. Cancer 2011, 11, 426. 21562580 10.1038/nrc3066

[20] A. M. Aravanis , M. Lee , R. D. Klausner , Cell 2017, 168, 571. 28187279 10.1016/j.cell.2017.01.030

[21] A. M. Newman , S. V. Bratman , J. To , J. F. Wynne , N. C. W. Eclov , L. A. Modlin , C. L. Liu , J. W. Neal , H. A. Wakelee , R. E. Merritt , J. B. Shrager , B. W. Loo Jr. , A. A. Alizadeh , M. Diehn , Nat. Med. 2014, 20, 548. 24705333 10.1038/nm.3519 PMC4016134

[22] F. Mouliere , D. Chandrananda , A. M. Piskorz , E. K. Moore , J. Morris , L. B. Ahlborn , R. Mair , T. Goranova , F. Marass , K. Heider , J. C. M. Wan , A. Supernat , I. Hudecova , I. Gounaris , S. Ros , M. Jimenez‐Linan , J. Garcia‐Corbacho , K. Patel , O. Østrup , S. Murphy , M. D. Eldridge , D. Gale , G. D. Stewart , J. Burge , W. N. Cooper , M. S. van der Heijden , C. E. Massie , C. Watts , P. Corrie , S. Pacey , K. M. Brindle , R. D. Baird , M. Mau‐Sørensen , C. A. Parkinson , C. G. Smith , J. D. Brenton , N. Rosenfeld , Sci. Transl. Med. 2018, 10, eaat4921. 30404863 10.1126/scitranslmed.aat4921 PMC6483061

[23] H. R. Underhill , J. O. Kitzman , S. Hellwig , N. C. Welker , R. Daza , D. N. Baker , K. M. Gligorich , R. C. Rostomily , M. P. Bronner , J. Shendure , PLOS Genet. 2016, 12, e1006162. 27428049 10.1371/journal.pgen.1006162 PMC4948782

[24] G. Leszinski , J. Lehner , U. Gezer , S. Holdenrieder , In Vivo 2014, 28, 299. 24815830

[25] M. Ivanov , A. Baranova , T. Butler , P. Spellman , V. Mileyko , BMC Genomics 2015, 16, S1. 10.1186/1471-2164-16-S13-S1 PMC4686799 26693644

[26] A. R. Thierry , F. Molina (CNRS), US 9580755, 2012.

[27] A. R. Thierry , C. Sanchez (INSERM), EU n°17306721.6, 2017.

[28] H. Tang , E. T. Donnell , Accid. Anal. Prev. 2019, 132, 105274. 31446099 10.1016/j.aap.2019.105274

[29] S. S. Panesar , R. N. D'Souza , F.‐C. Yeh , J. C. Fernandez‐Miranda , World Neurosurg.: X 2019, 2, 100012. 31218287 10.1016/j.wnsx.2019.100012 PMC6581022

[30] G. Wichmann , C. Gaede , S. Melzer , J. Bocsi , S. Henger , C. Engel , K. Wirkner , J. R. Wenning , T. Wald , J. Freitag , M. Willner , M. Kolb , S. Wiegand , M. Löffler , A. Dietz , A. Tárnok , Cancers 2019, 11, 814. 10.3390/cancers11060814 PMC6628584 31212819

[31] J. T. Jordan , D. E. McNiel , Psychiatry Res. 2018, 268, 317. 30096659 10.1016/j.psychres.2018.07.040

[32] Q. Q. Tiet , Y. E. Leyva , R. H. Moos , S. M. Frayne , L. Osterberg , B. Smith , JAMA Intern. Med. 2015, 175, 1371. 26075352 10.1001/jamainternmed.2015.2438

[33] R. Meddeb , Z. A. A. Dache , S. Thezenas , A. Otandault , R. Tanos , B. Pastor , C. Sanchez , J. Azzi , G. Tousch , S. Azan , C. Mollevi , A. Adenis , S. El Messaoudi , P. Blache , A. R. Thierry , Sci. Rep. 2019 2019, 9, 5220. 10.1038/s41598-019-41593-4 PMC6435718 30914716

[34] Y. Xu , Y. Song , J. Chang , X. Zhou , Q. Qi , X. Tian , M. Li , X. Zeng , M. Xu , W. Zhang , D. S. Cram , J. Liu , Eur. J. Clin. Invest. 2018, 48, e13015. 30079480 10.1111/eci.13015

[35] D. J. Dwivedi , L. J. Toltl , L. L. Swystun , J. Pogue , K.‐L. Liaw , J. I. Weitz , D. J. Cook , A. E. Fox‐Robichaud , P. C. Liaw , Crit. Care 2012, 16, R151. 22889177 10.1186/cc11466 PMC3580740

[36] L. Wang , L. Xie , Q. Zhang , X. Cai , Y. Tang , L. Wang , T. Hang , J. Liu , J. Gong , Coron. Artery Dis. 2015, 26, 296. 25714070 10.1097/MCA.0000000000000231 PMC4415965

[37] S. Tug , S. Helmig , E. R. Deichmann , A. Schmeier‐Jürchott , E. Wagner , T. Zimmermann , M. Radsak , M. Giacca , P. Simon , Exerc. Immunol. Rev. 2015, 21, 164. 25826002

[38] F. Mouliere , S. El Messaoudi , C. Gongora , A.‐S. Guedj , B. Robert , M. Del Rio , F. Molina , P.‐J. Lamy , E. Lopez‐Crapez , M. Mathonnet , M. Ychou , D. Pezet , A. R. Thierry , Transl. Oncol. 2013, 6, 319. 23730412 10.1593/tlo.12445 PMC3660801

[39] P. van der Leest , E. Schuuring , Mol. Oncol. 2020, 14, 487. 32017376 10.1002/1878-0261.12646 PMC7053232

[40] S. Cristiano , A. Leal , J. Phallen , J. Fiksel , V. Adleff , D. C. Bruhm , S. Ø. Jensen , J. E. Medina , C. Hruban , J. R. White , D. N. Palsgrove , N. Niknafs , V. Anagnostou , P. Forde , J. Naidoo , K. Marrone , J. Brahmer , B. D. Woodward , H. Husain , K. L. van Rooijen , M.‐B. W. Ørntoft , A. H. Madsen , C. J. H. van de Velde , M. Verheij , A. Cats , C. J. A. Punt , G. R. Vink , N. C. T. van Grieken , M. Koopman , R. J. A. Fijneman , J. S. Johansen , H. J. Nielsen , G. A. Meijer , C. L. Andersen , R. B. Scharpf , V. E. Velculescu , Nature 2019, 570, 385. 31142840 10.1038/s41586-019-1272-6 PMC6774252

[41] P. Jiang , C. W. M. Chan , K. C. A. Chan , S. H. Cheng , J. Wong , V. W.‐S. Wong , G. L. H. Wong , S. L. Chan , T. S. K. Mok , H. L. Y. Chan , P. B. S. Lai , R. W. K. Chiu , Y. M. D. Lo , Proc. Natl. Acad. Sci. USA 2015, 112, E1317. 25646427 10.1073/pnas.1500076112 PMC4372002

[42] R. Meddeb , E. Pisareva , A. R. Thierry , Clin. Chem. 2019, 65, 623. 30792266 10.1373/clinchem.2018.298323

[43] S. El Messaoudi , F. Rolet , F. Mouliere , A. R. Thierry , Clin. Chim. Acta 2013, 424, 222. 23727028 10.1016/j.cca.2013.05.022

[44] R. W. Platt , J. A. Hanley , H. Yang , Stat. Med. 2000, 19, 313. 10649298 10.1002/(sici)1097-0258(20000215)19:3<313::aid-sim370>3.0.co;2-k

[45] L. Breiman , Classification and Regression Trees, Routledge, New York 2017.

