# Supplementary material for: Machine Learning‐Assisted Evaluation of Circulating DNA Quantitative Analysis for Cancer Screening
Source: Adv Sci (Weinh). 2020 Jul 29;7(18):2000486. doi: 10.1002/advs.202000486 (PMC7509651; doi:10.1002/advs.202000486)
Supplement: Supplementary file 1 — Supporting Information [file ADVS-7-2000486-s001.pdf]

Copyright WILEY-VCH Verlag GmbH & Co. KGaA, 69469 Weinheim, Germany, 2020.

ADVANCED SCIENCE
Open Access

Supporting Information

for *Adv. Sci.*, DOI: 10.1002/advs.202000486

Machine Learning-Assisted Evaluation of Circulating DNA Quantitative Analysis for Cancer Screening

*Rita Tanos, Guillaume Tosato, Amaelle Otandault, Zahra Al Amir Dache, Laurence Pique Lasorsa, Geoffroy Tousch, Safia El Messaoudi, Romain Meddeb, Mona Diab Assaf, Marc Ychou, Stanislas Du Manoir, Denis Pezet, Johan Gagnière, Pierre-Emmanuel Colombo, William Jacot, Eric Assénat, Marie Dupuy, Antoine Adenis, Thibault Mazard, Caroline Mollevi, José Maria Sayagués, Jacques Colinge, and Alain R. Thierry**

Copyright WILEY-VCH Verlag GmbH & Co. KGaA, 69469 Weinheim, Germany, 2020.

Supporting Information

Title: Machine Learning Assisted Evaluation of Circulating DNA Quantitative Analysis for Cancer Screening

*Author(s), and Corresponding Author(s)**
*Rita Tanos, Guillaume Tosato, Amaelle Otandault, Zahra Al Amir Dache, Laurence Pique Lasorsa, Geoffroy Tousch, Safia El Messaoudi, Romain Meddeb, Mona Diab Assaf , Marc Ychou, Stanislas Du Manoir, Denis Pezet, Johan Gagnière, Pierre-Emmanuel Colombo, William Jacot , Eric Assénat, Marie Dupuy, Antoine Adenis, Thibault Mazard, Caroline Mollevi, José Maria Sayagués, Jacques Colinge, Alain R. Thierry**

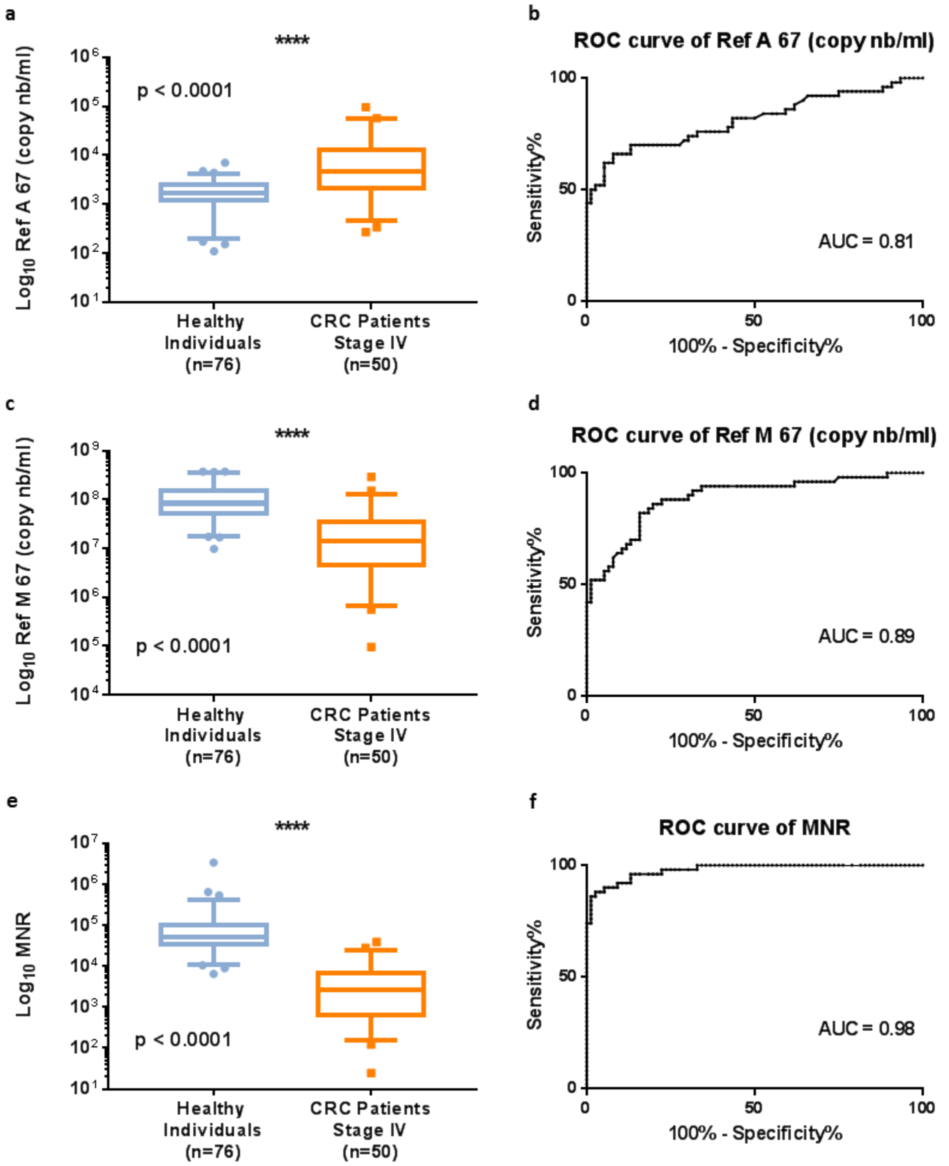

Figure S1. Efficiency of total nuclear and mitochondrial cfDNA amount to discriminate cancer and healthy patients in an exploratory cohort of 76 healthy individuals and 50 CRC patients of stage IV. **a.** Box plot of the total nuclear cfDNA concentration (Ref A 67 copy number/ml plasma) for healthy and stage IV CRC patients. **b.** ROC curve for Ref A 67 between healthy and stage IV CRC patients. **c.** Box plot of the total mitochondrial cfDNA concentration (Ref M 67 copy number/ml plasma) for healthy and stage IV CRC patients. **d.** Receiver operating characteristics (ROC) curve for Ref M 67 between healthy and stage IV CRC patients. **e.** Box Plot of the mitochondrial to nuclear DNA concentration ratio (MNR) for healthy and stage IV CRC patients. **f.** ROC curve for MNR between healthy and stage IV CRC patients.

The non-parametric Wilcoxon-Mann-Whitney test was used to compare the different parameters. The box plot whiskers represent the minimal and maximal value; p: p-value, probability value; ROC: receiver operating characteristics; AUC: area under curve; MNR: mitochondrial to nuclear ratio.

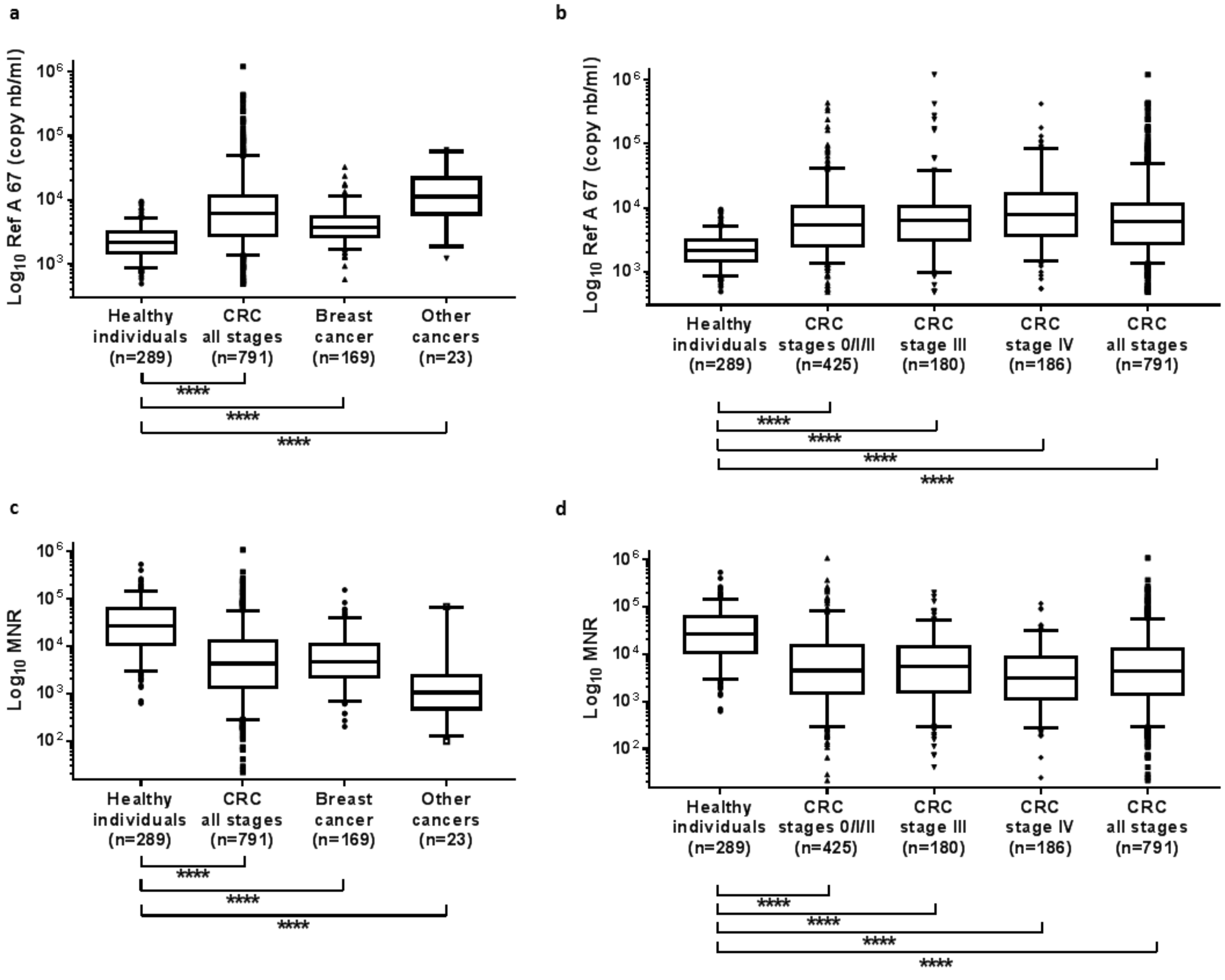

Figure S2. Ref A 67 and MNR evaluation in the plasma of healthy individuals and cancer patients. **a.** Box plot of the total nuclear cfDNA concentration (Ref A 67 copy number/ml of plasma) for healthy individuals and patients with different cancer types. **b.** Box plot of Ref A 67 for different stages of CRC. **c.** Box plot of the mitochondrial to nuclear DNA concentration ratio (MNR) for healthy individuals and patients with different cancer types. **d.** Box plot of MNR for different stages of CRC.

The non-parametric Wilcoxon-Mann-Whitney test was used to compare the different parameters. The box plot whiskers represent the 5th and 95th percentiles; **** correspond to a p-value < 0.0001.

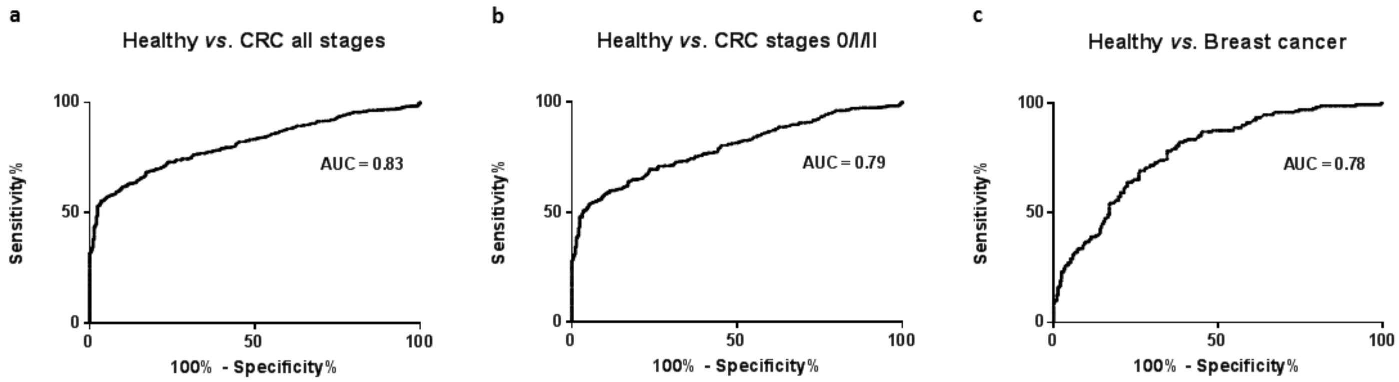

Figure S3. ROC curve of the Ref A 67 (copy nb/ml plasma) parameter for healthy individuals *vs*. **a.** CRC patients of all stages, **b.** early stages CRC patients (0/I/II), and **c.** breast cancer patients.

AUC: area under curve; ROC: receiver operating characteristics

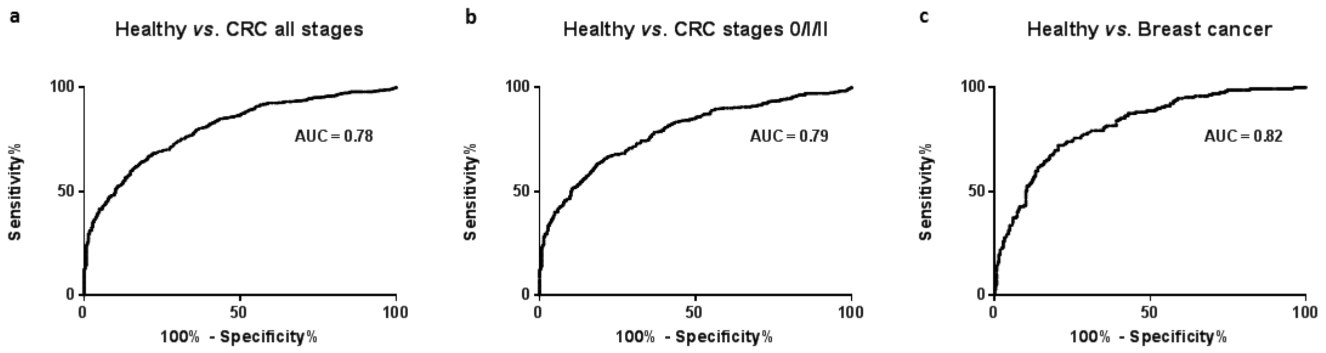

Figure S4. ROC curve of the MNR parameter for healthy individuals *vs*. **A.** CRC patients all stages, **B.** early stages CRC patients (0/I/II), and **C.** breast cancer patients.

MNR: mitochondrial to nuclear ratio; AUC: area under curve; ROC: Receiver operating characteristics

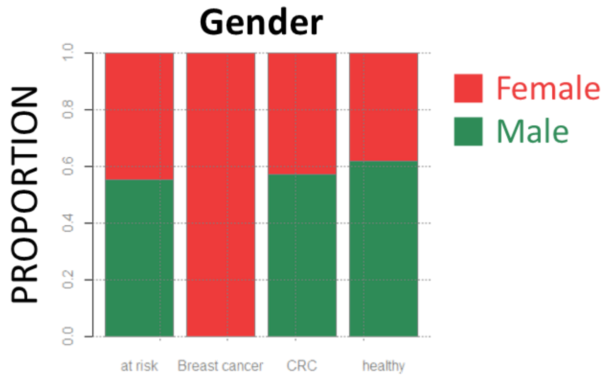

Figure S5. Gender distribution among healthy individuals and CRC patients.

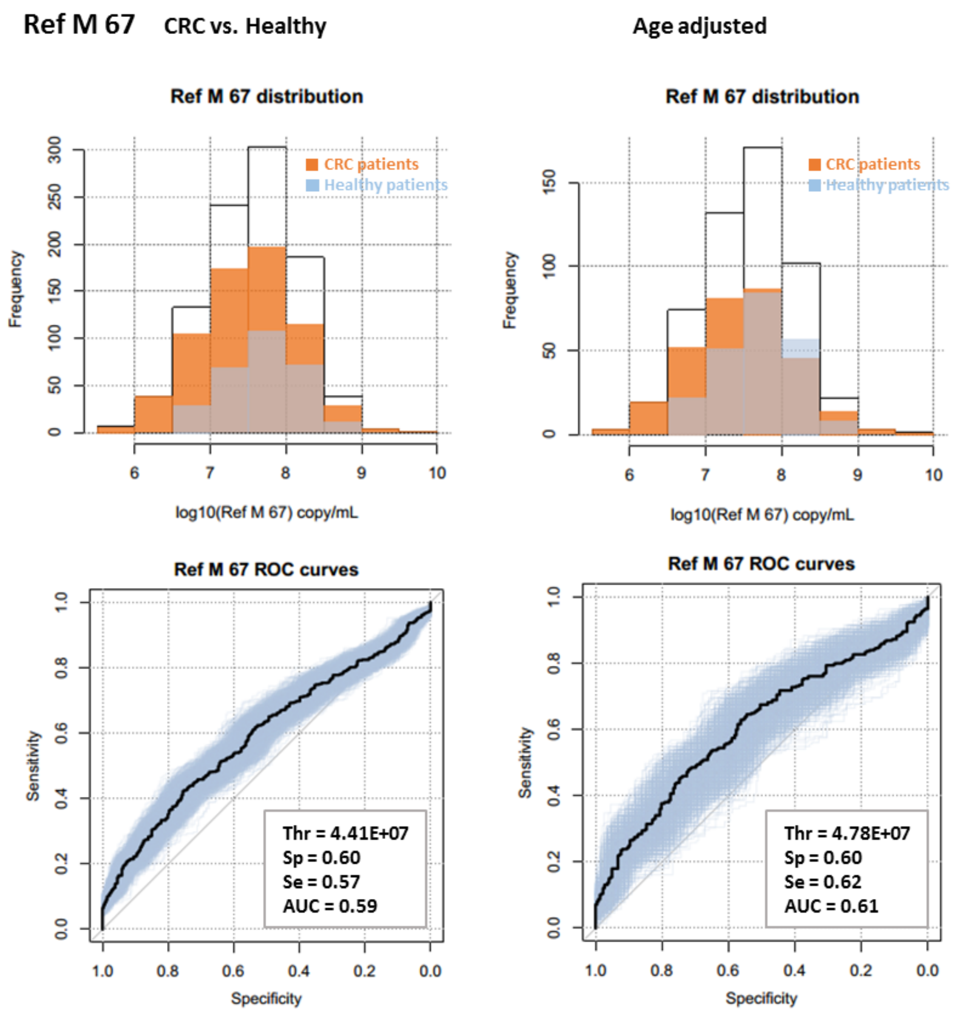

Figure S6. Distribution and ROC curves of the Ref M 67 (copy nb/ml) parameter among all patients (white), in CRC patients (orange) and healthy individuals (blue) before and after age adjustment.

Ref M 67: total mitochondrial cfDNA concentration; ROC: receiver operating characteristics; Thr: threshold; Sp: specificity; Se: sensitivity; AUC: Area under curve.

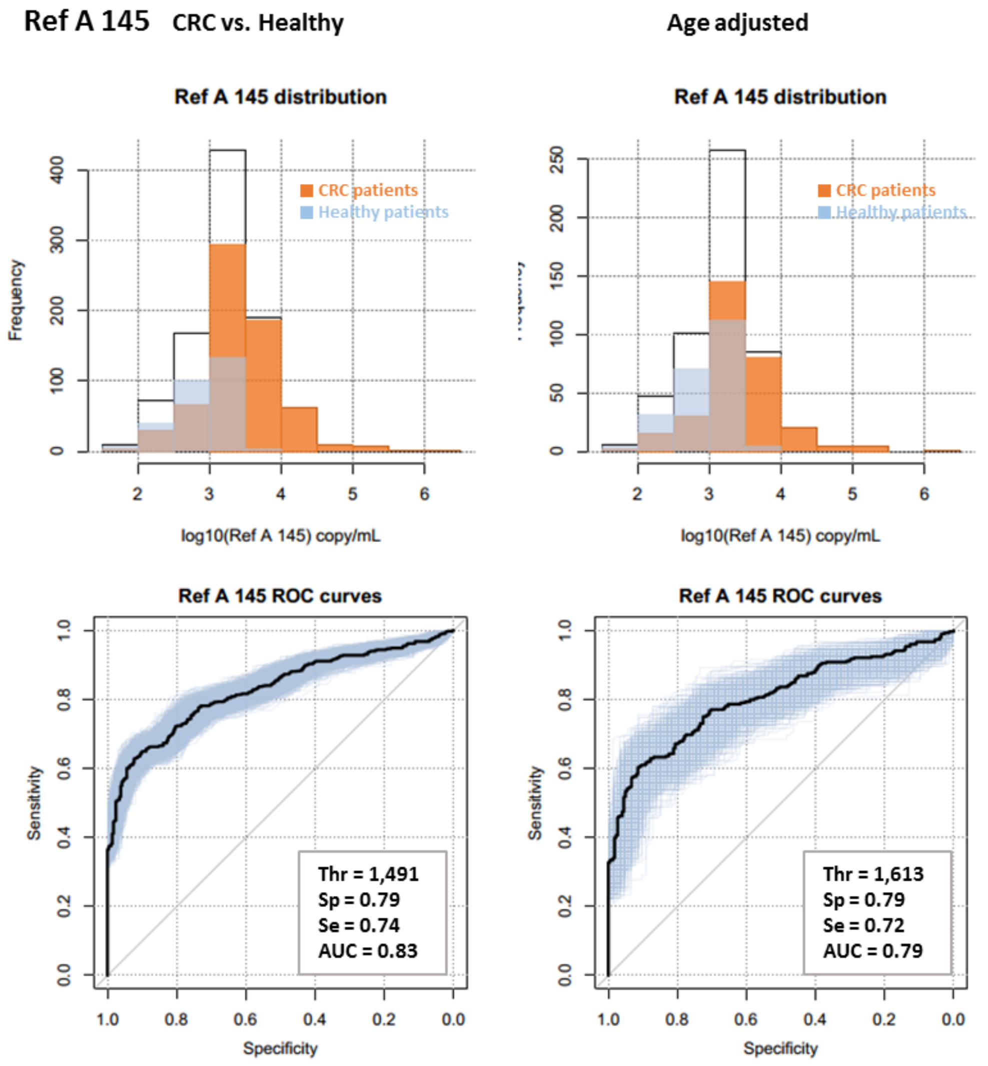

Figure S7. Distribution and ROC curves of the Ref A 145 (copy nb/ml) parameter among all patients (white), in CRC patients (orange) and healthy individuals (blue) before and after age adjustment.

Ref A 145: nuclear cfDNA concentration of the fragments with a size ≥ 145 base pairs; ROC: receiver operating characteristics; Thr: threshold; Sp: specificity; Se: sensitivity; AUC: area under curve.

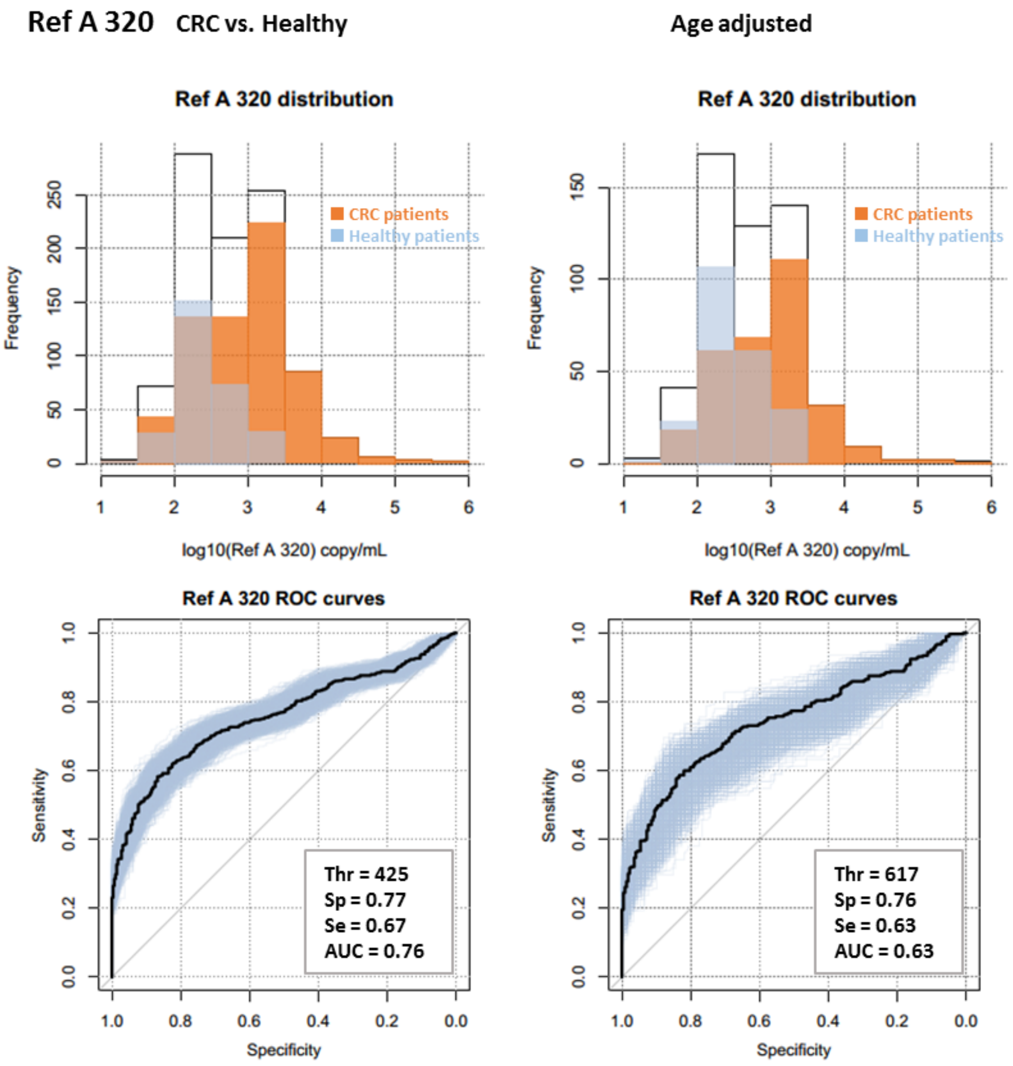

Figure S8. Distribution and ROC curves of the Ref A 320 (copy nb/ml) parameter among all patients (white), in CRC patients (orange) and healthy individuals (blue) before and after age adjustment.

Ref A 320: nuclear cfDNA concentration of the fragments with a size ≥ 320 base pairs; ROC: receiver operating characteristics; Thr: threshold; Sp: specificity; Se: sensitivity; AUC: Area under curve.

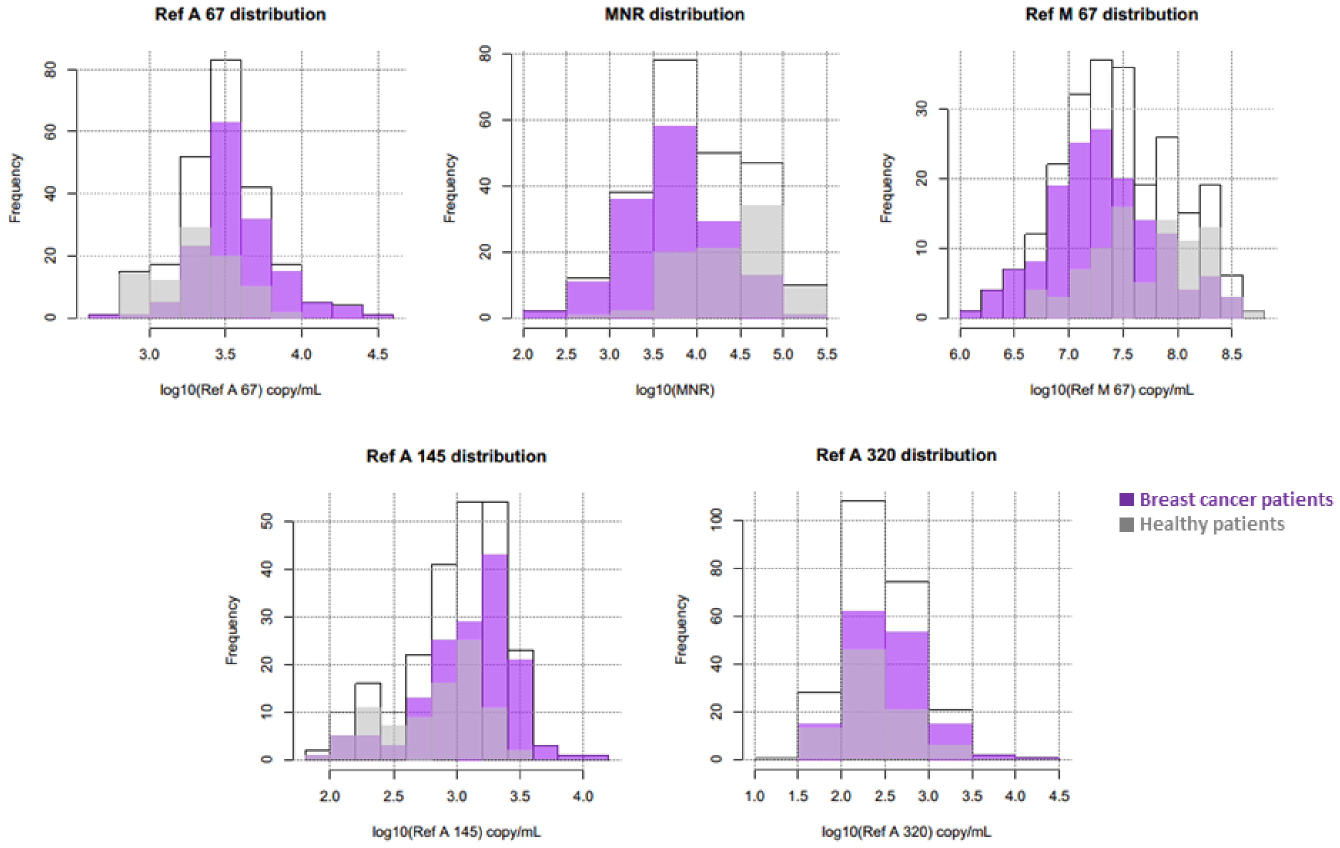

Figure S9. Distribution of each measured parameter among all patients (white), in breast cancer patients (purple) and healthy individuals (grey).

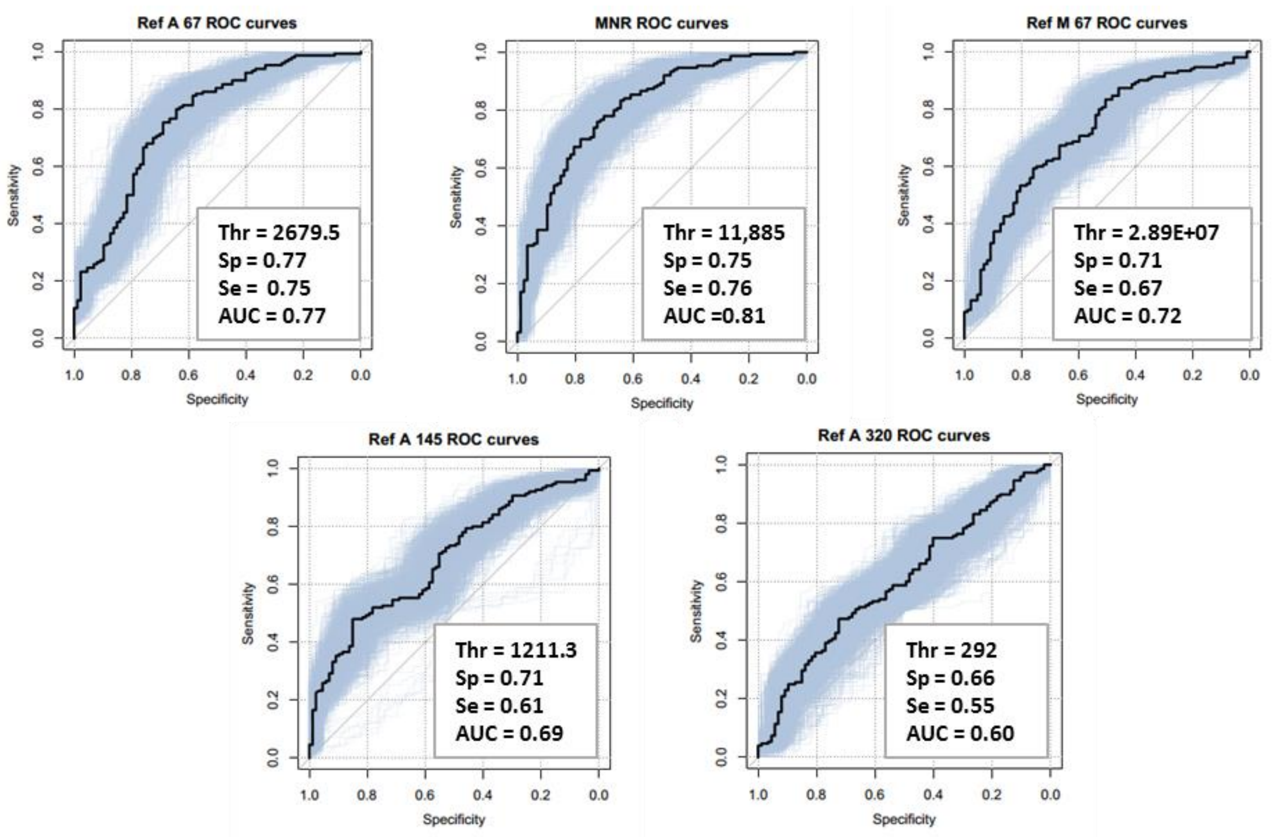

Figure S10. ROC curves of each measured parameter for the breast cancer cohort after 2,000 resampling for empirical bootstrapped estimation to build parameter confidence intervals. The original curve is presented in black.

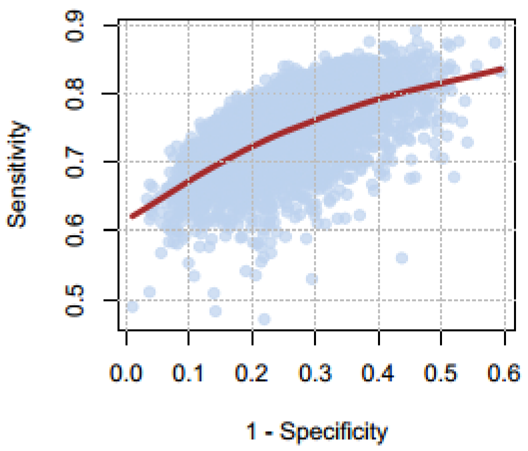

Figure S11. Plot of sensitivities and specificities obtained by creating multiple trees with resampling and cross-validation and its loess curve (in red).

Table S1. Different cancer and normal cell lines tested

	Type	Cell line
**Cancer cell lines (n=14)**	Colon	SW620<br>SW480<br>CaCo2
	Burkitt lymphoma	Ramos<br>BJAB
	Lung	H1975
	Prostate	VCAP<br>22rV1<br>DU145<br>LNCAP<br>PC3
	Breast	SUM159<br>MDA 468<br>R2shP53
**Normal cell lines (n=5)**	human foreskin fibroblasts	HFF
	human skin fibroblasts	CDC45K
	human mammary fibroblasts	R2
	human lung fibroblasts	IMR 90 A
	human normal hepatocytes	LWFD

Table S2. Descriptive statistics of the colorectal cancer cohort.

	**Colorectal Cancer Patients**		**Healthy Individuals**		
	**used**	entire cohort	**used**	entire cohort	
	**665**	794	**287**	289	
**Variable**	**Frequency**	**Proportion**	**Frequency**	**Proportion**	**p value**
Gender - female	283	42.60%	109	40.00%	0.698
	**Mean**	**Side Deviance**	**Mean**	**Side Deviance**	**p value**
Age (years)	70.4	11.7	43.4	14.0	<0.00001
Ref A 67 (copy/mL)	17456.5	62933.2	2473.4	1476.5	<0.00001
Ref A 145 (copy/mL)	8805.8	49244.8	1021.5	699.0	<0.00001
Ref A 320 (copy/mL)	5772.5	40694.0	392.4	431.9	<0.00001
Ref M 67 (copy/mL)	33052000.0	218748965.5	57092000.0	87677092.9	0.00002
MNR (Ref M 67 / Ref A 67)	16354.5	50162.3	45396.3	57714.4	<0.00001

Descriptive statistics of the colorectal cancer cohort after removing patients with missing values for the parameters of interest. Qualitative variables (frequency and proportion) are presented with the p-value produced by a two sample proportions test with continuity correction. Continuous variables (mean and standard deviation) are presented with the p-value produced by an unpaired two-sample non-parametric one-sided Mann-Whitney test with normal approximation (n>50).

Table S3. Estimation of the thresholds used for each node of the decision tree.

Tree localization		Ponctual estimation			95% Confidence interval	
Node	Parameter	**Median**	Mean	Sd	low	high
		**CRC Decision Tree**				
1	Ref A 67	**5348**	5218.1	572.5	3616	5818
2	MNR	**13450**	17440	10032	3020	34560
3	Ref A 145	**1388**	1507.1	388.8	451	2104
4.1	Ref A 67	**4259**	3987.7	505.6	2692	4312
4.2	Ref M 67	**8661000**	9388360	3734777	6807000	21270000
5	MNR	**6726**	5874.5	1844.6	2744	8644
		**Breast Cancer Decision Tree**				
1	MNR	**19450**	22792.5	11088.6	7468	37830
2.1	Ref A 67	**2487**	2455.8	291.5	1855	2928
2.2	Ref A 145	**1845**	1883.9	330	1398	2494
3	Ref A 145	**1226**	1193.2	360.4	410.9	1765
4	MNR	**5385**	6930.5	3017.4	3724	13330

Confidence intervals were obtained using the empirical bootstrap method on 2,000 independent resampling.

Table S4. Descriptive statistics of the breast cancer cohort.

	**Breast Cancer Patients**		**Healthy Individuals**		
	N= 169		N= 109		
	**mean**	**S.D.**	**mean**	**S.D.**	**p-value**
**Age (years)**	49.4	11.9	40.7	14.1	<0.00001
**Ref A 67 (copy/mL)**	4629.7	4040.9	2411.1	1460.9	<0.00001
**Ref A 145 (copy/mL)**	1664.1	1428.0	954.4	672.8	<0.00001
**Ref A 320 (copy/mL)**	636.6	1497.1	340.1	374.7	0.00059
**Ref M 67 (copy/mL)**	18104000.0	62578589.7	54296000.0	85962478.6	<0.00001
**MNR (Ref M 67 / Ref A 67)**	11223.9	17749.4	45204.7	47141.6	<0.00001

Descriptive statistics of the breast cancer cohort after removing patients with missing values for the parameters of interest. The variables are presented with their mean, standard deviation-, and the p-value produced by a two-sample non-parametric one-sided Mann-Whitney test with normal approximation (n >50) between breast cancer and healthy patients.

Table S5. ROC curves results from the breast cancer cohort with the use of an empirical bootstrap method to build confidence intervals.

	Threshold		Specificity		Sensitivity		AUC	
**Parameter**	**Value**	IC95%	**Value**	IC95%	**Value**	IC95%	**Value**	IC95%
**Ref A 67**	**2679.5**	[2431.3 - 3123.7]	**0.77**	[0.61 - 0.81]	**0.75**	[0.66 - 0.86]	**0.77**	[0.71 - 0.83]
**MNR**	**11884.8**	[4321.9 - 12869.4]	**0.75**	[0.66 - 0.85]	**0.76**	[0.66 - 0.85]	**0.81**	[0.76 - 0.87]
**Ref A 145**	**1211.3**	[868.9 - 1593.5]	**0.71**	[0.53 - 0.89]	**0.61**	[0.44 - 0.76]	**0.69**	[0.62 - 0.76]
**Ref A 320**	**291.6**	[226.8 - 432.2]	**0.66**	[0.52 - 0.86]	**0.55**	[0.34 - 0.65]	**0.60**	[0.53 - 0.70]
**Ref M 67**	**2.89e+07**	[1.78e+06 – 3.81e+07]	**0.71**	[0.59 - 0.85]	**0.67**	[0.50 - 0.77]	**0.72**	[0.66 - 0.88]

2,000 resampling have been done for the estimations. Parameter estimations for each variable on the breast cancer cohort were based on the distance between the corresponding ROC curve and the point (Se=1; 1-Sp=0), with the use of an empirical bootstrap method to build their respective confidence interval. These estimations were obtained after patient age pairing.
Se: sensitivity, Sp: specificity; AUC: area under curve.

Table S6. Prediction results of other cancer types using the tree obtained on the CRC and breast cancer cohort.

	Colorectal Cancer TREE prediction		Breast Cancer TREE prediction	
	Cancer	Not Cancer	Cancer	Not Cancer
ADP	2	1	3	0
HCC	18	0	18	0
Lymphoma	1	0	1	0
Ovarian cancer	0	1	0	1

Contingency table of predictive status after applying the decision trees built using both the colorectal cancer cohort and the breast cancer cohort.

Table S7. Table obtained using regression trees based on age resampling of patients from the CRC cohort.

Parameter	Estimation	IC low	IC high	Best
Sensitivity	**0.742**	0.65	0.851	0.788
Specificity	**0.746**	0.595	0.93	0.835

A training set of 206 patients was used from the 310 obtained at each resampling. The resulting tree was applied to the remaining patients, and the sensitivity and specificity of the cross-validation results were calculated. Confidence intervals were built by an empirical bootstrapping method on 2,000 resampling.

Table S8. Logistic model results concerning possible interaction between age and the different parameters.

	Univariate		Adjusted on age			Interaction with age		
**Parameters**	**Value**	**p-value**	**Value**	**p-value**	**age p-value**	**estimation**	**p-value**	**interaction p-value**
Ref A 67 (copy/mL)	5.19E-04	<0.00001	4.48E-04	<0.00001	<0.00001	1.19E-03	<0.00001	<0.00001
Ref M 67 (copy/mL)	1.43E-10	0.723	6.95E-10	0.212	<0.00001	5.30E-09	0.17	0.2
Ref A 320 (copy/mL)	0.001412	<0.00001	1.16E-03	<0.00001	<0.00001	2.57E-03	0.0123	0.1549
Ref A 145 (copy/mL)	0.0011382	<0.00001	9.96E-04	<0.00001	<0.00001	2.49E-03	0.000655	0.031487
MNR (Ref M 67 / Ref A 67)	-1.64E-05	<0.00001	-5.63E-06	0.026	<0.00001	-2.78E-05	0.134	0.212

Logistic parameter estimation and their associated p-value, used to evaluate the effect of each measured parameter on colorectal cancer probability in the cohort, and the effect of age. First, the effect of the parameter alone was evaluated in a single univariate logistic model. Second, a multivariate model was built using age as an adjusted covariable. Finally, a multivariate logistic model was built, with age and the interaction between age and the measured parameter as adjusted covariables.

Table S9. Characteristics of the primers selected to study nuclear and mitochondrial cfDNA.

HUMAN SEQUENCES				
**Gene**	**PRIMER NAME**	**Orientation**	**SEQUENCE 5' - 3'**	**AMPLICON SIZE (bp)**
**MITOCHONDRIAL DNA**				
MT-CO3	MIT MT-CO3 F	SENSE	GACCCACCAATCACATGC	67
	MIT MT-CO3 R 67	ANTISENSE	TGAGAGGGCCCCTGTTAG	
**NUCLEAR DNA**				
KRAS	KRAS B2 inv k	ANTISENSE	CCCTGACATACTCCCAAGGA	
	KRAS B1 inv k	SENSE	CCTTGGGTTTCAAGTTATATG	67
	KRAS 145 E	SENSE	GATAAAGGTTTCTCTGACCA	145
	KRAS A1 inv k	SENSE	GCCTGCTGAAAATGACTGA	320
BRAF	BRAF A1	SENSE	TTATTGACTCTAAGAGGAAAGATGAA	105
	BRAF A2	ANTISENSE	GAGCAAGCATTATGAAGAGTTTAGG	

MURINE SEQUENCES				
**Gene**	**PRIMER NAME**	**Orientation**	**SEQUENCE 5' - 3'**	**AMPLICON SIZE (bp)**
**MITOCHONDRIAL DNA**				
MT-CO1	MUMTCO1 F	SENSE	GTCCCACTAATAATCGGAGC	114
	MUMT CO1 REV C	ANTISENSE	TGCTTCTACTATTGATGATGC	
**NUCLEAR DNA**				
KRAS	KRAS 63-382 Mf	SENSE	AAGAGTGAAGACCCGTGTGC	63
	KRAS 63 Mr	ANTISENSE	GGAGAACAAGCACCCAACAG	
